# Identification of a New Lipoprotein Export Signal in Gram-Negative Bacteria

**DOI:** 10.1128/mBio.01232-16

**Published:** 2016-10-25

**Authors:** Frédéric Lauber, Guy Richard Cornelis, Francesco Renzi

**Affiliations:** Département de Biologie, Unité de Recherche en Biologie des Microorganismes (URBM), Université de Namur, Namur, Belgium

## Abstract

Bacteria of the phylum *Bacteroidetes*, including commensal organisms and opportunistic pathogens, harbor abundant surface-exposed multiprotein membrane complexes (Sus-like systems) involved in carbohydrate acquisition. These complexes have been mostly linked to commensalism, and in some instances, they have also been shown to play a role in pathogenesis. Sus-like systems are mainly composed of lipoproteins anchored to the outer membrane and facing the external milieu. This lipoprotein localization is uncommon in most studied Gram-negative bacteria, while it is widespread in *Bacteroidetes*. Little is known about how these complexes assemble and particularly about how lipoproteins reach the bacterial surface. Here, by bioinformatic analyses, we identify a lipoprotein export signal (LES) at the N termini of surface-exposed lipoproteins of the human pathogen *Capnocytophaga canimorsus* corresponding to K-(D/E)_2_ or Q-A-(D/E)_2_. We show that, when introduced in sialidase SiaC, an intracellular lipoprotein, this signal is sufficient to target the protein to the cell surface. Mutational analysis of the LES in this reporter system showed that the amino acid composition, position of the signal sequence, and global charge are critical for lipoprotein surface transport. These findings were further confirmed by the analysis of the LES of mucinase MucG, a naturally surface-exposed *C. canimorsus* lipoprotein. Furthermore, we identify a LES in *Bacteroides fragilis* and *Flavobacterium johnsoniae* surface lipoproteins that allow *C. canimorsus* surface protein exposure, thus suggesting that *Bacteroidetes* share a new bacterial lipoprotein export pathway that flips lipoproteins across the outer membrane.

## IMPORTANCE

Bacteria of the phylum *Bacteroidetes* are important human commensals and pathogens. Understanding their biology is therefore a key question for human health. A main feature of these bacteria is the presence of abundant lipoproteins at their surface that play a role in nutrient acquisition. To date, the underlying mechanism of lipoprotein transport is unknown. We show for the first time that *Bacteroidetes* surface lipoproteins share an N-terminal signal that drives surface localization. The localization and overall negative charge of the lipoprotein export signal (LES) are crucial for its role. Overall, our findings provide the first evidence that *Bacteroidetes* are endowed with a new bacterial lipoprotein export pathway that flips lipoproteins across the outer membrane.

## INTRODUCTION

Among Gram-negative bacteria, the phylum *Bacteroidetes* is composed of a large diversity of organisms widely distributed in the environment. Some are saprophytes such as *Flavobacteria*, found in soil (1) and aquatic environments (2), while others are commensal organisms of animals. Among the commensal organisms, *Bacteroides* spp. are common members of the intestinal flora where they play a major role in gut homeostasis (3–7), while *Capnocytophaga* and *Porphyromonas* spp. are part of the oral flora (8, 9). *Bacteroides fragilis*, a commensal of the human intestine, and *Capnocytophaga canimorsus*, a common member of the dog oral flora can cause severe systemic human infections (10–15), while *Porphyromonas gingivalis* causes severe periodontal diseases (8). The wide distribution of these organisms reflects their high adaptability, partially due to their vast array of glycosylhydrolases allowing them to degrade nearly all types of carbohydrates they can encounter (7, 16–19). Interestingly, these enzymes are often surface-exposed lipoproteins and are part of multiprotein outer membrane (OM) complexes devoted to nutrient acquisition. These complexes, facing the outside environment (20, 21), are encoded in genetic regions named polysaccharide utilization loci (PUL) (19) that represent a hallmark of this phylum.

To date, most studies have focused on identifying and characterizing the functions of these *Bacteroidetes* surface complexes (5, 7, 16–18, 22, 23), but little is known about how they assemble (24) and particularly about how lipoproteins reach the bacterial surface. In Gram-negative *Proteobacteria*, lipoprotein synthesis and transport have been well studied in model organisms such as *Escherichia coli* (25). Lipoproteins are first synthesized as a precursor in the cytoplasm before their translocation to the periplasm via the Sec (26, 27) or Tat (28–30) machinery. This recognition is mediated by the N-terminally located signal peptide II (31), which contains a conserved cysteine residue critical for the subsequent steps of maturation (32, 33). After crossing the inner membrane (IM), lipoprotein precursors remain anchored to the periplasmic side of the IM where they are then processed by three enzymes, rendering a final triacylated lipoprotein (34–37). Lipoproteins destined to be inserted into the OM are transported through the aqueous environment of the periplasm via the dedicated Lol (localization of lipoproteins) transport machinery, composed of five proteins, LolA, -B, -C, -D, and -E (25, 38). In *Proteobacteria*, most OM lipoproteins are inserted in the inner leaflet of the OM and thus face the periplasm. The surface localization of OM lipoproteins in *Bacteroidetes* thus implies the existence of a yet unknown dedicated recognition and transport mechanism.

The present study deals with the reference strain *C. canimorsus* 5 (39), which encodes 13 PUL. Three of them were recently shown to play critical roles in the biology and pathogenesis of this bacterium (40–42). We address the question of how lipoproteins are targeted to the bacterial surface. We identify a signal sequence (lipoprotein export signal [LES]) present at the N termini of surface-exposed lipoproteins, and we show that this signal is sufficient to target an intracellular lipoprotein to the cell surface. We extend our findings to other *Bacteroidetes* species, namely, *Flavobacterium johnsoniae* and *Bacteroides fragilis*, identifying their specific LESs, thus showing that they share a new bacterial lipoprotein export pathway that flips lipoproteins across the outer membrane.

## RESULTS

### *In silico* identification of a putative lipoprotein export signal.

**FIG 1:**
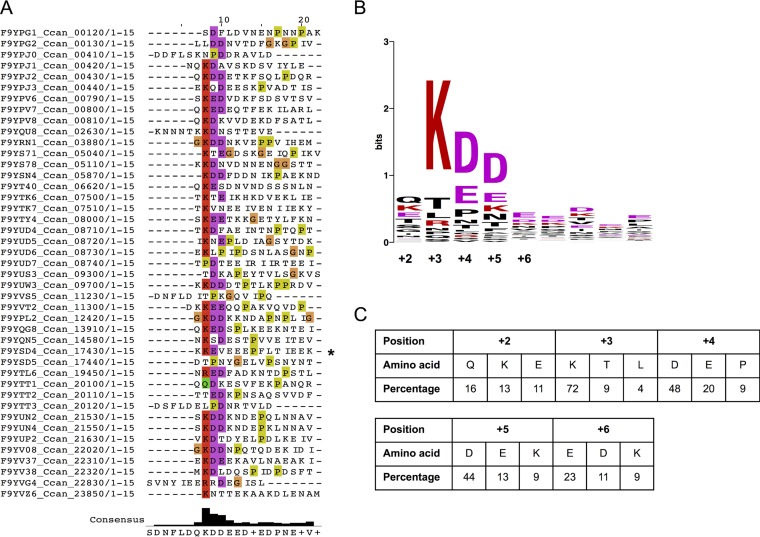
Alignment of *C. canimorsus* surface-exposed lipoproteins reveals the presence of an N-terminal conserved motif. (A) MAFFT alignment of the first 15 N-terminal amino acids of mature surface-exposed lipoproteins. The first invariant cysteine residue of each sequence was removed before the alignment was performed. Highly conserved residues are indicated according to the Clustal color code (R and K in red; D and E in magenta; P in yellow; G in orange; Q, N, S, and T in green, C in pink; A, I, L, M, F, W, and V in blue; H and Y in cyan) (63). MucG (Ccan_17430) is indicated by an asterisk. The derived consensus sequence is shown below the sequence alignment. (B) Generated WebLogo of the consensus sequence determined in panel A. Positions relative to the +1 cysteine are indicated below the WebLogo. Charged residues are indicated in color. The color code is the same as that used in panel A. (C) Amino acid frequency for each position of the consensus sequence, expressed as a percentage. The three most represented amino acids for each position are shown.

In order to see whether a specific amino acid motif would be responsible for the targeting of lipoproteins to the bacterial surface, we examined in detail the sequences of the lipoproteins detected at the surface of *Capnocytophaga canimorsus* strain 5 (17). When aligning the mature lipoproteins, a lysine (K), followed by either an aspartate (D) or a glutamate (E) residue, appeared to be conserved in close proximity to the N-terminal cysteine at position +1 (see Fig. S1 in the supplemental material). This was refined by a second alignment considering only the 15 N-terminal residues of the mature lipoprotein and excluding the invariant first cysteine (Fig. 1A). The resulting consensus motif corresponded to Q-K-D-D-E, located between positions +2 and +6 (Fig. 1B) showing conservation of 16, 72, 48, 44, and 23%, respectively (Fig. 1C). In order to determine whether this motif is specific to the surface-exposed lipoproteins, the same analysis was performed on OM lipoproteins facing the periplasm (17). No highly conserved residues were identified in this set of proteins (see Fig. S2 in the supplemental material), suggesting that the QKDDE consensus motif could indeed be a bona fide lipoprotein export signal (LES).

### The LES leads to surface localization of the periplasmic lipoprotein sialidase.

**FIG 2:**
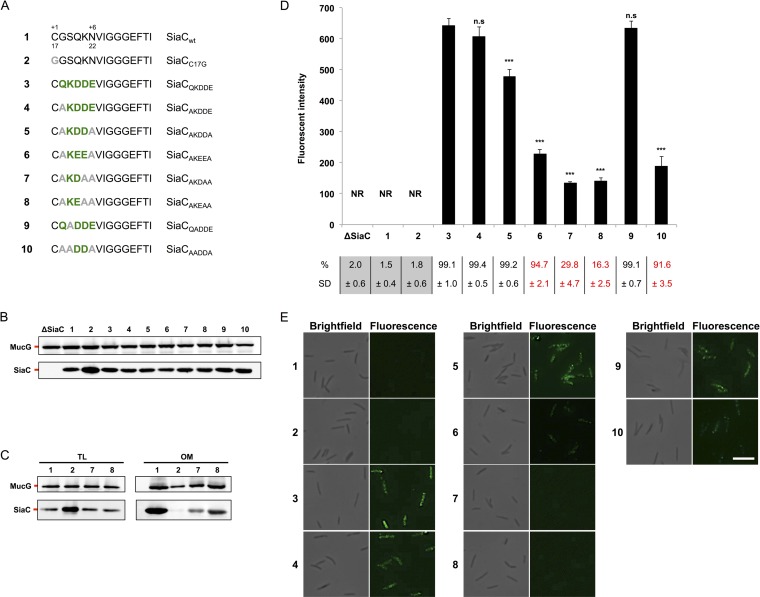
The LES allows SiaC surface exposure. (A) Wild-type (wt) SiaC and consensus sequence mutant constructs. Amino acids derived from the consensus sequence (green boldface) and point mutations (gray boldface) are indicated. The SiaC constructs are referred to by the boldface numbers shown in panel A in panels B to E. (B) Detection of SiaC by Western blot analysis of total cell extracts of strains expressing the SiaC constructs shown in panel A. Expression of MucG was monitored as a loading control. (C) Detection of SiaC by Western blot analysis of total lysates (TL) and outer membrane (OM) fractions of bacteria expressing different SiaC constructs. Expression of MucG was monitored as a loading control. (D) Quantification of SiaC surface exposure by flow cytometry of live cells labeled with anti-SiaC serum. The fluorescence intensity of stained cells only is shown (NR, not relevant). The averages from at least three independent experiments are shown. Error bars represent 1 standard deviation from the mean. Values that are significantly different (*P* ≤ 0.001) from the value for reference construct 3 are indicated (***). Values that are not significantly different (n.s) from the value for reference construct 3 are indicated. The percentage of stained cells and standard deviation (SD) are indicated below the bar graph. Values below the detection limit (≤2.5%) are shown on a gray background. Values for strains with a statistically significant lower stained population are shown in red (*P* ≤ 0.001 compared to reference construct 3). (E) Immunofluorescence microscopy images of bacteria labeled with anti-SiaC serum. Bar, 5 µm.

To verify this hypothesis, we introduced the QKDDE motif in the sequence of the *C. canimorsus* sialidase (SiaC), an OM lipoprotein that faces the periplasm (42, 43). SiaC harboring the LES (SiaC_+2QKDDE+6_) (Fig. 2A and B) was detected at the bacterial surface by immunolabeling, followed by flow cytometry and microscopy (Fig. 2D and E). In contrast, wild-type (wt) SiaC and the soluble SiaC_C17G_ variant were not detected. This indicated that the addition of the consensus motif to an OM periplasmic lipoprotein is sufficient to drive its transport to the bacterial surface and hence that this consensus motif represents a LES.

### Determination of the minimal consensus motif allowing surface localization of sialidase.

We next determined the minimal sequence required to constitute a functional LES. We first replaced the least conserved amino acids of the LES, namely, the +2 Q and +6 E, by alanine residues, generating constructs SiaC_+2AKDDE+6_ and SiaC_+2AKDDA+6_ (Fig. 2A). After monitoring protein expression (Fig. 2B), immunolabeling showed that both constructs localized to the bacterial surface (Fig. 2D and E), although to a lower extent than SiaC_+2QKDDE+6_ did, thus indicating that the KDD motif is sufficient to target lipoproteins to the cell surface. We then tested whether glutamate was able to functionally replace aspartate (SiaC_+2AKEEA+6_) (Fig. 2A), since both residues were enriched in the alignment (Fig. 1C). Replacing the two aspartate residues with two glutamate residues did not prevent surface localization but led to a clear reduction of fluorescence (Fig. 2D and E), in line with the lower conservation of glutamate at positions +4 and +5 (Fig. 1C), showing that in *C. canimorsus* surface lipoproteins, aspartate is preferred over glutamate.

We then generated two SiaC constructs harboring only either KD or KE (SiaC_+2AKDAA+6_ and SiaC_+2AKEAA+6_) (Fig. 2A), but these two pairs of residues alone turned out to be very weak LESs since only 29.8% ± 4.7% (SiaC_+2AKDAA+6_) and 16.3% ± 2.5% (SiaC_+2AKEAA+6_) of the cells displayed the proteins at their surface (Fig. 2D). In addition, the fluorescence intensity was weak, 28.2 and 29.4%, respectively, of the intensity observed for the SiaC_+2AKDDA+6_ reference (Fig. 2D). In order to verify that these constructs were not impaired in their transport to the OM, we monitored their presence in isolated outer membrane fractions. Both mutant proteins were found to be anchored to the OM although at lower levels than the wt protein, in particular for the construct SiaC_+2AKDAA+6_, suggesting that these mutations could also impact to a minor extent OM localization of SiaC (Fig. 2C). Overall, these data supported our hypothesis that K-(D/E)_2_ represents the minimal LES. These findings also suggested that a functional LES might require an overall negative charge, supported by the fact that KDD is allowing efficient transport of SiaC to the surface, while only KD is not (Fig. 2D).

We then investigated the importance of the highly conserved lysine residue at position +3 of the LES (Fig. 2A). Unexpectedly, substitution of K alone (SiaC_+2QADDE+6_) had no impact on the display of SiaC at the bacterial surface (Fig. 2D and E). However, removal of both K and Q (SiaC_+2AADDA+6_) led to a decrease of fluorescence intensity of more than 60% compared to that of SiaC_+2AKDDA+6_. Since the glutamine residue itself was not found to be critical (SiaC_+2AKDDA+6_ [Fig. 2D]), we conclude that either the +2 Q or the +3 K is required to form a functional LES. Taken together, these data indicate that the minimal export motif allowing surface localization of SiaC is composed of only two negatively charged amino acids preceded by a positively charged or polar residue. On the basis of the consensus, we thus defined the minimal *C. canimorsus* LES as being K-(D/E)_2_ or Q-A-(D/E)_2_.

### Positional effect of the minimal LES on sialidase surface localization.

**FIG 3:**
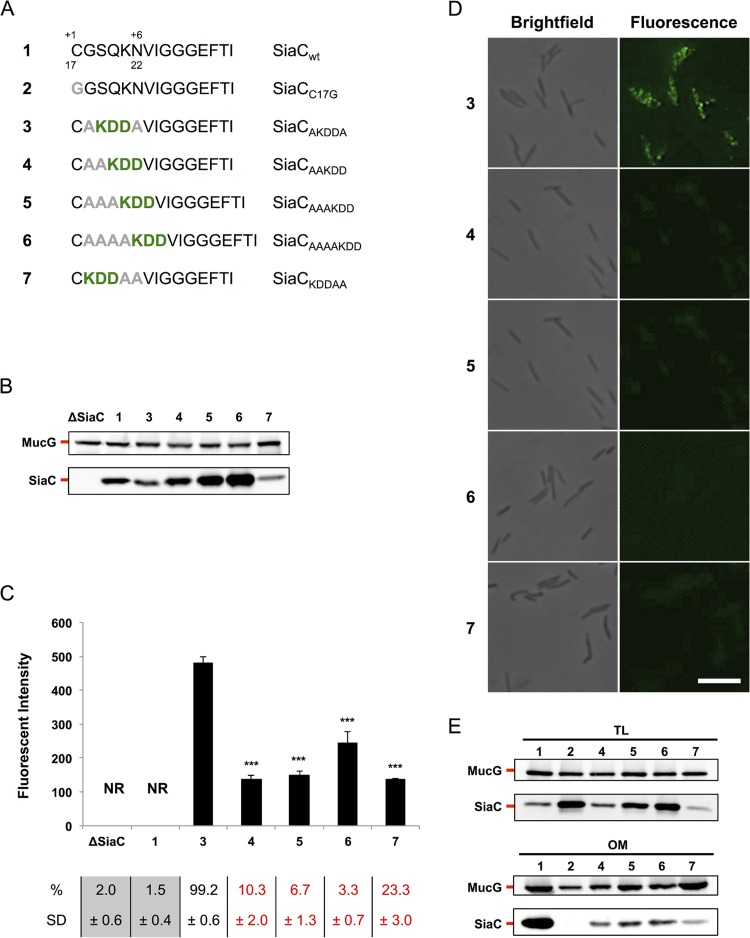
The position of the minimal LES is crucial for its function. (A) wt SiaC and consensus sequence mutant constructs. Amino acids derived from the consensus sequence (green boldface) and point mutations (gray boldface) are indicated. The SiaC constructs are referred to by the boldface numbers shown in panel A in panels B to E. (B) Detection of SiaC by Western blot analysis of total cell extracts of strains expressing the SiaC constructs shown in panel A. MucG expression was monitored as a loading control. (C) Quantification of SiaC surface exposure by flow cytometry of live cells labeled with anti-SiaC serum. The fluorescence intensity of stained cells only is shown (NR, not relevant). The averages from at least three independent experiments are shown. Error bars represent 1 standard deviation from the mean. Values that are significantly different (*P* ≤ 0.001) from the value for reference construct 3 are indicated (***). The percentage and standard deviation (SD) of stained cells are indicated below the bar graph. Values below the detection limit (≤2.5%) are shown on gray background. Values for strains with a statistically significant lower stained population are shown in red (*P* ≤ 0.001 compared to the value for the reference construct 3). (D) Immunofluorescence microscopy images of bacteria stained with anti-SiaC serum. Bar, 5 µm. (E) Western blot analysis of total lysates (TL) and outer membrane (OM) fractions of bacteria expressing different SiaC constructs. MucG expression was monitored as a loading control.

We next addressed the question of the importance of the position of the LES. The initial alignment showed that K is conserved mainly at position +3 (72%), to a lower extent at position +2 (13%), and is completely absent from position +4 (Fig. 1C). In contrast, D and E were conserved at positions +4, +5, and +6 (48, 44, and 11% for D and 20, 13, and 23% for E, respectively) and completely absent from position +3 (Fig. 1C). This suggested that not only the composition of the export signal could be crucial but also its position relative to the +1 cysteine. Therefore, we generated constructs in which the KDD motif was separated from the +1 cysteine by zero, two, three, or four alanine residues (Fig. 3A) and compared their surface localization to the construct in which the KDD motif is separated from the +1 cysteine by only one alanine residue (SiaC_+2AKDDA+6_). Although the four proteins were expressed (Fig. 3B), none of them were exported as efficiently as the one where only one alanine separated the KDD motif from the +1 cysteine (SiaC_+2AKDDA+6_) (Fig. 3C and D). All proteins were anchored to the OM, thus again indicating that only the last step of transport to the surface was affected by these mutations (Fig. 3E). The position of the K-(D/E)_2_ signal relative to the +1 cysteine is thus critical for the *C. canimorsus* LES, and the optimal sequence is C-X-K-(D/E)_2_-X.

### Characterization of the LES of the surface-exposed lipoprotein MucG.

Looking at the LESs of different *C. canimorsus* surface lipoproteins (Fig. 1), it appeared that some were quite divergent from the consensus. Among these is the LES of mucinase MucG (41) (Ccan_17430), KKEVEEE (Fig 1A; see Fig. S3A in the supplemental material). We first confirmed that MucG is indeed a surface-exposed lipoprotein (see Fig. S3 in the supplemental material), and then we tested whether this poorly conserved LES would drive the export of sialidase to the surface of *C. canimorsus*. We introduced the MucG LES, KKEVEEE, or part of this sequence, into SiaC, giving SiaC_+2KKEVE+6_, SiaC_+2KKEVEE+7_, and SiaC_+2KKEVEEE+8_ (see Fig. S4A and S4B in the supplemental material) and monitored their surface localization (see Fig. S4C and S4D in the supplemental material). SiaC_+2KKEVE+6_ was only poorly transported to the cell surface, while SiaC_+2KKEVEE+7_ and SiaC_+2KKEVEEE+8_ showed clear surface localization. Although the overall protein amount of SiaC_+2KKEVE+6_ was reduced, the protein appeared to be anchored to the OM (see Fig. S4E in the supplemental material). As the only difference between these constructs was the number of negatively charged amino acids in the LES, this strongly supported our initial findings that the LES requires an overall negative charge to drive transport of lipoproteins to the bacterial surface (Fig. 2).

**FIG 4:**
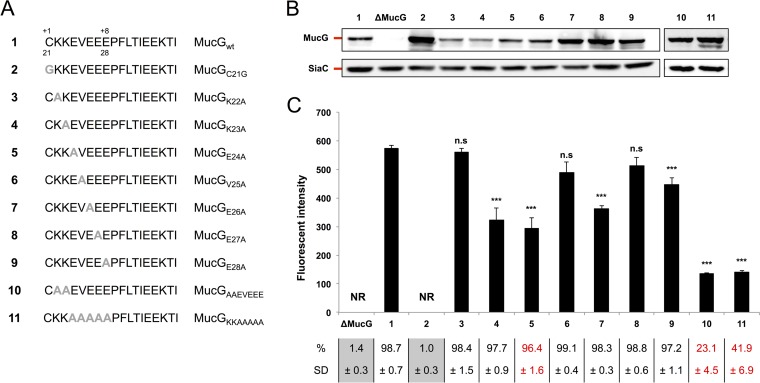
MucG LES mutational analysis. (A) wt MucG and mutant constructs. Point mutations are indicated in gray boldface type. The MucG constructs are referred to by the boldface numbers shown in panel A in panels B and C. (B) Detection of MucG by Western blot analysis of total cell extracts of strains expressing the MucG constructs shown in panel A. Expression of SiaC was monitored as a loading control. (C) Quantification of MucG surface exposure by flow cytometry of live cells labeled with anti-MucG serum. The fluorescence intensity of stained cells only is shown (NR, not relevant). The averages from at least three independent experiments are shown. Error bars represent 1 standard deviation from the mean. Values that are significantly different (*P* ≤ 0.001) from the value for reference construct 1 are indicated (***). Values that are not significantly different (n.s) from the value for reference construct 1 are indicated. The percentage and standard deviation (SD) of stained cells are indicated below the bar graph. Values below the detection limit (≤2.5%) are shown on a gray background. Values for strains with a statistically significant lower stained population are shown in red (*P* ≤ 0.001 compared to the value for reference construct 1).

We next wanted to study the MucG LES in its native background. To this aim, we systematically replaced residues 22 to 28 of the MucG LES by alanines (Fig. 4A). After verifying that all mutant proteins were expressed (Fig. 4B), we monitored the surface exposure of the MucG variants by flow cytometry (Fig. 4C). Replacing K22, V25, and E27 with alanine did not significantly alter surface exposure of MucG, while mutation of K23, E24, E26, or E28 resulted in a 25 to 50% decrease of surface exposure. None of these single mutations completely abolished surface localization, suggesting that the MucG motif is redundant, presumably due to the presence of two lysines and four glutamates. The mutation of one of those residues could therefore be compensated for by the presence of another one in close proximity, and indeed, all protein variants we generated harbor an overall negatively charged functional LES.

Because of this, we generated two additional constructs by mutating simultaneously either all negatively or all positively charged residues in the MucG LES (Fig. 4A). After having confirmed their correct expression (Fig. 4B), we analyzed their surface localization by flow cytometry (Fig. 4C). As expected, replacing the two lysine residues (MucG_AAEVEEE_) led to MucG surface exposure in only 23.1% ± 4.5% of the cells (Fig. 4C). Furthermore, the fluorescence intensity in this subset of cells was markedly decreased compared to the wt strain (23.8%), indicating that the efficiency of transport was also strongly affected in this subpopulation. This is in good agreement with our previous findings showing the importance of the K/Q residues for surface export (Fig. 2).

Similarly, MucG_KKAAAAA_ was surface localized in only 41.9% ± 6.9% of the cells (Fig. 4C), and the fluorescence intensity in this subpopulation was lower than the fluorescence intensity of the wt strain (24.5%). This is in agreement with our findings in SiaC that an overall negatively charged LES is critical for efficient surface localization.

By combining the data obtained from single and multiple alanine substitutions, the minimal LES for optimal MucG surface exposure appears to be X-K-(D/E)_3_ downstream from the +1 cysteine, hence resembling the LES deduced from previous experiments [X-K-(D/E)_2_-X] (Fig. 2 and 3; see Fig. S4 in the supplemental material).

### The LES is conserved in the *Bacteroidetes* phylum.

To determine whether the LES identified in *C. canimorsus* would be conserved in other *Bacteroidetes*, we took advantage of the recently published *B. fragilis* NCTC 9343 surfome study (44) and performed an *in silico* analysis on the N termini of the identified surface lipoproteins (see Fig. S5A in the supplemental material). We found an enrichment in negatively charged amino acids in close proximity to the +1 cysteine (SDDDD) (see Fig. S5A in the supplemental material). However, unlike the *C. canimorsus* LES, the aspartate residues were mostly located at positions +3 and +4 instead of positions +4 and +5. Additionally, this region was not enriched in positively charged amino acids at position +3 but harbored a polar serine residue at position +2. This sequence is different from the LES identified in *C. canimorsus*, but nevertheless, it is clearly similar to the *C. canimorsus* LES. Indeed, it starts with a polar residue followed by several negatively charged residues, and in *C. canimorsus*, the lysine residue could be replaced by an alanine provided that a glutamine was present at position +2 (Fig. 2D and E). We thus hypothesize that SDDDD represents the consensus LES of *B. fragilis*. We then searched for the LES of *Flavobacterium johnsoniae* UW101 that belongs to the same family as *C. canimorsus*, the *Flavobacteriaceae*. Since no surfome analysis has been performed on this bacterium, we recovered the sequences of all predicted SusD homologs (19), supposedly surface-exposed lipoproteins. We next aligned their N termini and derived the consensus sequence SDDFE (see Fig. S5B in the supplemental material). Interestingly, this motif seems closer to the LES of *B. fragilis* than to the *C. canimorsus* LES in the sense that it is enriched in a polar residue (S) rather than in a positively charged one. However, negatively charged amino acids are still predominant in this LES.

### The LESs from *B. fragilis* and *F. johnsoniae* are functional in *C. canimorsus*.

**FIG 5:**
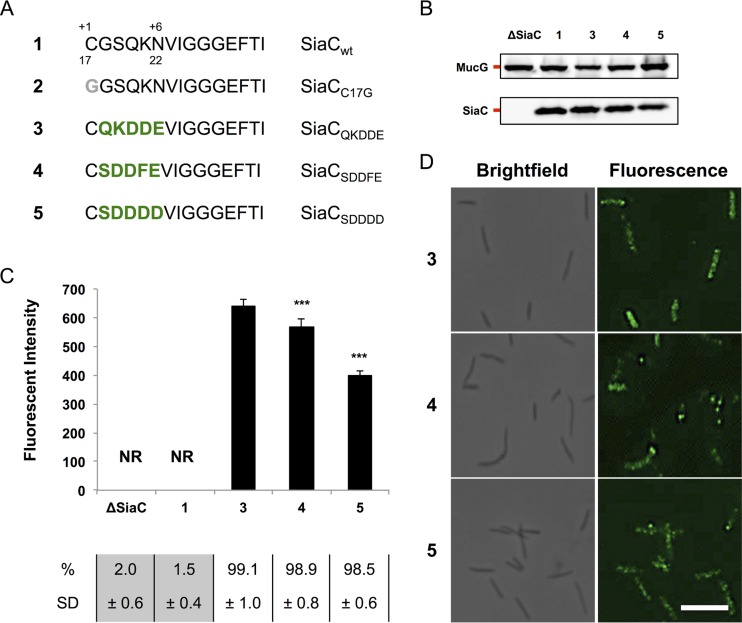
*B. fragilis* and *F. johnsoniae* LES allow SiaC surface localization. (A) wt SiaC and consensus sequence mutant constructs. Amino acids derived from the *B. fragilis* or *F. johnsoniae* consensus sequence (green boldface) and point mutations (gray boldface) are indicated. The SiaC constructs are referred to by the boldface numbers shown in panel A in panels B to D. (B) Detection of SiaC by Western blot analysis of total cell extracts of strains expressing the SiaC constructs shown in panel A. MucG expression was monitored as a loading control. (C) Quantification of SiaC surface exposure by flow cytometry of live cells labeled with anti-SiaC serum. The fluorescence intensity of stained cells only is shown (NR, not relevant). The averages from at least three independent experiments are shown. Error bars represent 1 standard deviation from the mean. Values that are significantly different (*P* ≤ 0.001) from the value for reference construct 3 are indicated (***). The percentage and standard deviation (SD) of stained cells are indicated below the bar graph. Values below the detection limit (≤2.5%) are shown on a gray background. (D) Immunofluorescence microscopy images of bacteria labeled with anti-SiaC serum. Bar, 5 µm.

To validate our findings, we tested whether the consensus sequences predicted for *B. fragilis* (SDDDD) and *F. johnsoniae* (SDDFE) would represent a functional LES in *C. canimorsus*. Both sequences were inserted in SiaC (Fig. 5A), and the recombinant proteins were tested in *C. canimorsus* (Fig. 5B). Both constructs were found to be surface localized (Fig. 5C and D), although at lower levels than SiaC harboring the *C. canimorsus* LES, indicating that the LESs from *Bacteroides* and *Flavobacteria* allow surface transport of lipoproteins in *Capnocytophaga*. Overall, these data confirm the evidence of a shared novel pathway for lipoprotein export in this phylum of Gram-negative bacteria.

## DISCUSSION

In conclusion, we show for the first time that surface-exposed lipoproteins of *Bacteroidetes* harbor a specific signal at their N terminus that drives their transport to the bacterial surface. In addition, we derived the canonical LES sequence that represents the most common choice of amino acid at each position for *C. canimorsus*, *B. fragilis*, and *F. johnsoniae*. For *C. canimorsus*, it is C-X-K-(D/E)_2_-X, where X can be any amino acid as long as the overall negative charge of the LES is maintained. Interestingly, this is different from what has been described in the *Spirochaetes Borrelia burgdorferi*. This bacterium, also harboring a high proportion of surface lipoproteins, seems to transport them to its surface by default without the requirement of a specific signal (45–47). This suggests that *Bacteroidetes* and *Spirochaetes* evolved different lipoprotein transport machineries and corresponding signaling pathways. The LES of *Bacteroidetes* is in direct proximity to the +1 cysteine, a region that acts as a Lol avoidance signal in *Proteobacteria* (48–50), thus indicating that the sorting rules distinguishing inner and outer membrane lipoproteins are also different in *Bacteroidetes*.

The discovery of the LES implies the existence of a novel export pathway in bacteria and represents the starting point for the identification of the machinery that allows surface lipoprotein localization. In this regard, it is interesting to note that *Bacteroidetes* do not carry genes that encode any homolog of LolB, the OM lipoprotein responsible for the insertion of lipoproteins into the inner leaflet of the OM in *E. coli* and most studied bacteria (51, 52). The function of LolB in *Bacteroidetes* might therefore be fulfilled by another protein or protein complex that would also be able to flip surface-exposed lipoproteins across the OM.

Recently, a novel lipoprotein export system has been discovered in the human pathogen *Neisseria meningitidis* (53). This bacterium displays several lipoproteins at the bacterial surface; these lipoproteins include the TbpA and HupA proteins that are involved in iron uptake from transferrin and hemoglobin, respectively (54, 55). Hooda et al. (53) have shown that TbpA is transported to the bacterial surface by an integral outer membrane protein named Slam1 (surface lipoprotein assembly modulator 1), while HupA is transported by the paralog Slam2. While homologs of Slam could be found in several *Proteobacteria* (53), we could not identify any homolog in *Bacteroidetes*, thus suggesting that in this phylum, lipoproteins are transported to the bacterial surface via a different mechanism. Furthermore, in *Neisseria*, no conserved signal sequence has so far been identified in surface-exposed lipoproteins, and the evidence that TbpA and HupA each require a specific Slam transporter suggests that in this bacterium, the recognition between the lipoprotein and the transporter is different from *Bacteroidetes*, where a common specific sequence would send the lipoproteins to the bacterial surface. We believe the discovery of the LES represents a step forward in understanding the complex biology of *Bacteroidetes*, composed of commensal organisms and opportunistic pathogens, and is the starting point for the identification of the machinery that allows localization of surface lipoproteins.

## MATERIALS AND METHODS

### Bacterial strains and growth conditions.

Bacterial strains used in this study are listed in Table S1 in the supplemental material. *Escherichia coli* strains were routinely grown in lysogeny broth (LB) at 37°C. *Capnocytophaga canimorsus* strains were routinely grown on heart infusion agar (Difco) supplemented with 5% sheep blood (Oxoid) plates (SB plates) for 2 days at 37°C in the presence of 5% CO_2_. To select for plasmids, antibiotics were added at the following concentrations: 100 µg/ml ampicillin (Amp) and 50 µg/ml kanamycin (Km) for *E. coli* and 10 µg/ml erythromycin (Em), 10 µg/ml cefoxitin (Cfx), and 20 µg/ml gentamicin (Gm) for *C. canimorsus*.

### Construction of *siaC* and *mucG* expression plasmids.

Plasmids and primers used in this study are listed in Tables S2 and S3 in the supplemental material, respectively*. siaC* (*Ccan_04790*) was amplified from 100 ng of *C. canimorsus* strain 5 genomic DNA with primers 4159 and 7696 using the Q5 high-fidelity DNA polymerase (catalog no. M0491S; New England Biolabs). The initial denaturation was performed at 98°C for 2 min, followed by 30 cycles of amplification (1 cycle consisting of 98°C for 30 s, 52°C for 30 s, and 72°C for 2 min) and finally 10 min at 72°C. After purification, the fragment was digested using NcoI and XhoI restriction enzymes and cloned into plasmid pMM47.A, yielding plasmid pFL117. *mucG* (*Ccan_17430*) was cloned in the same way except that primers 7182 and 7625 were used for amplification and the fragment was cloned into plasmid pPM5, yielding plasmid pFL43.

Site-specific point mutations were introduced by amplifying separately the N- and C-terminal part of each gene using forward and reverse primers harboring the desired mutations in their sequence in combination with primers 4159 and 7696 for *siaC* and primers 7182 and 7625 for *mucG*. Both PCR fragments were purified and then mixed in equal amounts for PCR using the PrimeStar HS DNA polymerase (catalog no. R010A; Takara). The initial denaturation step was performed at 98°C for 2 min, followed by 30 cycles of amplification (98°C for 10 s, 60°C for 5 s, and 72°C for 3 min 30 s) and finally 10 min at 72°C. Final PCR products were then cleaned, digested using NcoI and XhoI restriction enzymes, and cloned into plasmids pMM47.A and pPM5 for *siaC* and *mucG*, respectively. The incorporation of the desired point mutations in all inserts was confirmed by sequencing. Plasmids expressing *siaC* and *mucG* variants were transferred to *C. canimorsus* strain 5 *siaC* and *mucG* deletion strains, respectively, by electroporation (39).

### Immunofluorescence labeling for flow cytometry (fluorescence-activated cell sorting [FACS]) and microscopy (indirect immunofluorescence [IF]) analysis.

Bacteria grown for 2 days on SB plates were collected, washed once with phosphate-buffered saline (PBS), and resuspended in 1 ml PBS to an optical density at 600 nm (OD_600_) of 0.1. Five microliters of bacterial suspension (approximately 3 × 10^5^ bacteria) were used to inoculate 2.5 ml of Dulbecco modified Eagle medium (DMEM) (catalog no. 41965-039; Gibco) containing 10% heat-inactivated human serum (HIHS) in 12-well plates (catalog no. 665 180; Greiner Bio-One). Bacteria were harvested after 23 h of growth at 37°C in the presence of 5% CO_2_, washed twice with PBS, and resuspended in 1 ml PBS. The optical density at 600 nm of bacterial suspensions was measured, and approximately 3 × 10^7^ bacteria were collected for each strain. Bacteria were resuspended in 200 µl PBS containing 1% bovine serum albumin (BSA) (wt/vol) and incubated for 30 min at room temperature. Bacteria were then centrifuged, resuspended in 200 µl of a primary antibody dilution (rabbit anti-SiaC or rabbit anti-MucG antiserum), and incubated for 30 min at room temperature. Following centrifugation, bacterial cells were washed three times before being resuspended in 200 µl of a secondary antibody dilution (donkey anti-rabbit coupled to Alexa Fluor 488 [catalog no. A-21206; Invitrogen]) and incubated for 30 min at room temperature in the dark. Following centrifugation, bacteria were washed three times, resuspended in 200 µl of 4% paraformaldehyde (PFA) (wt/vol) and incubated for 15 min at room temperature in the dark. Finally, bacteria were centrifuged, washed once, and resuspended in 700 µl of PBS. For flow cytometry analysis, samples were directly analyzed with a BD FACSVerse (BD Biosciences), and data were processed with BD FACSuite (BD Biosciences). For microscopy analysis, labeled bacteria were added to the top of poly-l-lysine-coated coverslips and allowed to adhere for 30 min at room temperature. After removal of the bacterial suspension, the coverslips were washed three times, mounted upside down on glass slides, and allowed to dry overnight at room temperature in the dark. All microscopy images were captured with an Axioscop (Zeiss) microscope with an Orca-Flash 4.0 camera (Hamamatsu) and Zen 2012 software (Zeiss). For a control, samples were prepared in parallel as described above except that rabbit preimmunization serum was used for labeling.

### *In vivo* radiolabeling with [^3^H]palmitate, immunoprecipitation, and fluorography.

Bacteria were grown overnight as described above for immunofluorescence labeling, except that bacteria were grown in 5 ml medium in six-well plates (catalog no. 657 160; Greiner Bio-One). After 18 h of incubation, [9,10-^3^H]palmitic acid (32 Ci/mmol) (catalog no. NET043; PerkinElmer Life Sciences) was added to a final concentration of 50 µCi/ml, and incubation was continued for 6 h. Bacteria were then collected by centrifugation and washed two times with 1 ml PBS, and the pellets were stored at −20°C until further use. Pellets were resuspended in 300 µl PBS containing 1% Triton X-100 (catalog no. 28817.295; VWR) and vortexed 10 s to lyse bacteria. Lysates were centrifuged 2 min at 14,000 × *g*, and the supernatant was transferred to a new tube. MucG proteins were immunoprecipitated by the addition of 15 µl MucG antiserum for 90 min at room temperature with constant agitation. In parallel, 20 µl of protein A agarose slurry (catalog no. P3476; Sigma-Aldrich) was washed two times with 500 µl of wash buffer (0.1% Triton X-100 in PBS), saturated with 500 µl of 0.2% BSA (wt/vol) for 30 min, and washed again two times with wash buffer. The protein A agarose slurry was then added to the cell lysate, and incubation was continued for 30 min at room temperature with constant agitation. Samples were then centrifuged at 14,000 × *g* for 2 min, and the supernatant was discarded. Pellets were washed five times with 500 µl of wash buffer. Bound proteins were eluted by the addition of 50 µl SDS-PAGE buffer and heating for 10 min at 95°C. Samples were centrifuged again, and supernatants were carefully separated from the agarose beads and loaded on 10% SDS-polyacrylamide gels. After gel electrophoresis, the gels were fixed in a solution of isopropanol, water, and acetic acid (25:65:10) overnight and subsequently soaked for 30 min in Amplify (NAMP100; Amersham) solution. Gels were vacuum dried and exposed to SuperRX autoradiography film (Fuji) for 13 to 21 days until the desired signal strength was reached.

### Human salivary mucin degradation.

Fresh human saliva was collected from healthy volunteers and filter sterilized using 0.22-µm filters (Millipore). Bacteria grown for 2 days on SB plates were collected and washed once with PBS, and the solution was adjusted to an OD_600_ of 1. One hundred microliters of bacterial suspension (approximately 5 × 10^7^ bacteria) were then mixed with 100 µl of human saliva and incubated for 240 min at 37°C. For a negative control, 100 µl of saliva was incubated with 100 µl PBS. Samples were then centrifuged for 5 min at 13,000 ×  *g*, and the supernatants were carefully collected and loaded on 10% SDS-polyacrylamide gels. Mucin degradation was monitored by lectin staining with peanut agglutinin (PNA) (digoxigenin [DIG] glycan differentiation kit [catalog no. 11210238001; Roche]) according to the manufacturer’s instructions. Mucin degradation was estimated by loss or reduction of PNA staining compared to the negative control.

### Outer membrane protein purification.

Outer membrane proteins were isolated as described in references 44 and 56 with several modifications. All steps were carried out on ice unless stated otherwise. All sucrose concentrations are expressed as percentages (wt/vol) in 10 mM HEPES (pH 7.4). Bacteria collected from two plates were washed two times with 30 ml of 10 mM HEPES (pH 7.4) before being resuspended in 4.5 ml of 10% sucrose. Bacterial cells were then disrupted by two passages through a French press at 35,000 lb/in^2^. The lysate was collected and centrifuged for 10 min at 16,500 × *g* to remove insoluble material. The crude cell extract was then layered on top of a sucrose step gradient composed of 1.33 ml of 70% sucrose and 6 ml of 37% sucrose and centrifuged at 100,000 × *g* (28,000 rpm) for 70 min at 4°C in an SW41 Ti rotor. The yellow material above the 37% sucrose solution and at the 10%/37% interface, corresponding to soluble and enriched inner membrane proteins, was collected and diluted to 7 ml with 10 mM HEPES (pH 7.4). The high-density band at the 37%/70% interface, corresponding to enriched outer membrane proteins, was collected and diluted to 7 ml with 10 mM HEPES (pH 7.4). Membranes from both fractions were then centrifuged at 320,000 × *g* (68,000 rpm) for 90 min at 4°C in a 70.1 Ti rotor. The supernatant of the yellow material fraction, corresponding to soluble proteins, was transferred to a fresh tube and stored at −20°C. The pellet of the same tube, corresponding to a mixture of inner and outer membrane fractions, was resuspended in 1 ml of 40% sucrose and stored at −20°C. The supernatant of the outer membrane protein band was discarded, and the pellet was resuspended in 7 ml of 10 mM HEPES (pH 7.4) containing 1% Sarkosyl (catalog no. L5777; Sigma-Aldrich) and incubated at room temperature for 30 min with constant agitation. The outer membrane fraction was then centrifuged at 320,000 ×  *g* for 60 min at 4°C in a 70.1 Ti rotor, resuspended in 7 ml of 100 mM Na_2_CO_3_ (pH 11), and incubated at 4°C for 20 min with constant agitation. The outer membrane fraction was then centrifuged, washed with 7 ml unbuffered 40 mM Tris, and centrifuged again. Finally, the purified outer membrane was resuspended in 200 to 400 µl unbuffered 40 mM Tris and stored at −20°C. Protein concentration of all fractions was assessed using the Bio-Rad protein assay dye reagent (catalog no. 5000006; Bio-Rad) according to the manufacturer’s instructions. Samples (1 to 2 µg) of total protein from whole-cell lysates and outer membrane fractions were loaded onto 12% SDS-polyacrylamide gels. After gel electrophoresis, proteins were transferred onto nitrocellulose membranes and analyzed by Western blotting.

### Multiple-sequence alignment of lipoproteins.

The sequences of 41 lipoproteins previously identified as being part of the surface proteome of *C. canimorsus* strain 5 (17) were retrieved from the UniProt database (57) (release 2015_12). Additionally, two *C. canimorsus* 5 proteins (UniProt accession no. F9YSD4 and F9YTT3) detected at the bacterial surface but predicted to harbor a signal peptidase I (SPI) signal were reanalyzed with the PATRIC database (58) and found to possess an SPII signal and thus considered lipoproteins, resulting in a final list of 43 surface-exposed predicted lipoproteins (see Table S4 in the supplemental material). The SPII cleavage site of each protein was then predicted using the LipoP software (59) (1.0 server, default settings), showing that all proteins possess one clear SPII cleavage site. Accordingly, protein sequences were trimmed to their predicted mature form. Lists corresponding to either full-length protein sequences or 15 amino acids downstream of the +1 cysteine were generated. Data sets were then submitted to multiple-sequence alignment using the MAFFT online tool (60) (version 7.268, default settings), and the output was analyzed using the Jalview software (61) (version 2.9.0b2). The final consensus sequence logo was drawn using WebLogo (62) (version 2.8.2, default settings). The sequences of the 17 *C. canimorsus* outer membrane lipoproteins presumably facing the periplasm (17) were processed in the same way (Table S5). The sequences of the 22 previously identified proteinase K-sensitive *Bacteroides fragilis* NCTC 9343 surface-exposed lipoproteins (44) were processed in the same way (Table S5). Forty-two *Flavobacterium johnsoniae* UW101 predicted SusD-like lipoproteins were identified in the PUL database (PULDB) of the CAZY database (19), and the corresponding sequences were extracted from the UniProt database and processed as described above (Table S5).

### Statistical analysis.

All data are presented as means ± standard deviations (SD). Statistical analyses were done by one-way analysis of variance (ANOVA), followed by Bonferroni test using the GraphPad Prism version 5.00 for Windows (GraphPad Software, La Jolla, CA, USA). A *P* value of ≤0.05 was considered statistically significant.

## SUPPLEMENTAL MATERIAL

Figure S1  Multiple-sequence alignment of full-length *C. canimorsus* surface lipoproteins. MAFFT alignment was performed on mature surface-exposed lipoproteins. Only the N-terminal region showing the conserved K-(D/E) motif is displayed. Highly conserved residues are indicated according to the Clustal color code (R and K in red; D and E in magenta; P in yellow; G in orange; Q, N, S, and T in green; C in pink; A, I, L, M, F, W, and V in blue; H and Y in cyan). The derived consensus sequence is shown below the alignment. Download Figure S1, TIF file, 2.6 MB

Figure S2  Multiple-sequence alignment of *C. canimorsus* periplasmic outer membrane lipoproteins. MAFFT alignment was performed on the first 15 N-terminal amino acids of intracellular OM lipoproteins. The first invariant cysteine residue of each sequence was removed before the alignment was performed. Highly conserved residues are indicated according to the Clustal color code (see the legend to Fig. S1 in the supplemental material). The derived consensus sequence is shown below. SiaC (Ccan_04790) is indicated by an asterisk. Download Figure S2, TIF file, 1.1 MB

Figure S3  MucG is a surface-exposed lipoprotein. (A) MucG domain annotation. Predicted structural domains are indicated by gray boxes. Amino acid positions are indicated above the map. The predicted LES is shown below the map. (B) Western blot analysis (top) and fluorography (bottom) of the elution fraction of MucG immunoprecipitation of [^3^H]palmitate-labeled bacteria. MucG is lipidated in the wt strain and the Δ*mucG* strain expressing MucG, but not in the Δ*mucG* strain expressing MucG_C21G_ in which the predicted site of lipidation is mutated, showing that MucG is a lipoprotein. Rabbit IgG hc is the heavy chain of the rabbit MucG antiserum present in the analyzed elution fraction. The low-molecular-weight band in the MucG strain likely represents a truncated MucG form due to overexpression. This band being radiolabeled indicates that the truncation takes place at the C terminus of MucG. The two low-molecular-weight bands in the MucG_C21G_ mutant likely represent two different MucG truncated forms that are generated when the protein overexpressed is not lipidated and periplasmic. (C) MucG detection by Western blot analysis of total cell lysates (TL) and outer membrane (OM) fractions of bacteria expressing different MucG constructs. MucG, but not the soluble MucG_C21G_, is detected in the OM fraction, showing that MucG is a bona fide OM lipoprotein. SiaC expression was monitored as a loading control. (D) Quantification of MucG surface exposure by flow cytometry of live cells labeled with anti-MucG serum. The fluorescence intensity of stained cells only is shown (NR, not relevant). The averages from at least three independent experiments are shown. Error bars represent 1 standard deviation from the mean. The percentage and standard deviation (SD) of stained cells are indicated below the bar graph. Values below the detection limit (≤2.5%) are shown on a gray background. (E) Immunofluorescence microscopy images of bacteria labeled with anti-MucG serum. Bar, 5 µm. (F) Detection of mucin by PNA lectin staining of human saliva following incubation with bacteria expressing different MucG constructs performed as described in reference 17. Untreated saliva was used as a negative control. Reduction of PNA staining indicates mucin degradation by surface-localized MucG. Download Figure S3, TIF file, 2.4 MB

Figure S4  The MucG LES allows SiaC surface localization. (A) wt SiaC and MucG consensus sequence mutant constructs. Amino acids derived from the consensus or MucG LES (green boldface) and point mutations (gray boldface) are indicated. The SiaC constructs are referred to by the boldface numbers shown in panel A in panels B to E. (B) Detection of SiaC by Western blot analysis of total cell extracts of strains expressing the SiaC constructs shown in panel A. MucG expression was monitored as a loading control. (C) Immunofluorescence microscopy images of bacteria labeled with anti-SiaC serum. Bar, 5 µm. (D) Quantification of SiaC surface exposure by flow cytometry of live cells labeled with anti-SiaC serum. The fluorescence intensity of stained cells only is shown (NR, not relevant). The averages from at least three independent experiments are shown. Error bars represent 1 standard deviation from the mean. Values that are significantly different (*P* ≤ 0.001) from the value for reference construct 3 are indicated (***). The percentage and standard deviation (SD) of stained cells are indicated below the bar graph. Values below the detection limit (≤2.5%) are shown on a gray background, and values for strains with a statistically significant lower stained population are in red (*P* ≤ 0.001 compared to the value for reference construct 3). (E) Western blot analysis of total lysates (TL) and outer membrane (OM) fractions of bacteria expressing different SiaC constructs. MucG expression was monitored as a loading control. Download Figure S4, TIF file, 2.9 MB

Figure S5  Multiple-sequence alignment of *B. fragilis* and *F. johnsoniae* surface lipoproteins. (A) MAFFT alignment of the first 16 N-terminal amino acids of proteinase K-sensitive *B. fragilis* lipoproteins. (B) MAFFT alignment of the first 16 N-terminal amino acids of SusD-like *F. johnsoniae* lipoproteins. Highly conserved residues are indicated according to the Clustal color code (see the legend to Fig. S1 in the supplemental material). Corresponding WebLogo and amino acid frequencies are indicated below. Download Figure S5, TIF file, 2.7 MB

Table S1  Bacterial strains used in this study. Table S1, DOCX file, 0.1 MB

Table S2  Plasmids used in this study. Footnote *a* on Vectors indicates that selection markers for *C. canimorsus* are within parentheses. Table S2, DOCX file, 0.1 MB

Table S3  Oligonucleotides used in this study. Footnote *a* on Restriction indicates that the restriction sites are underlined in the sequence column. Table S3, DOCX file, 0.1 MB

Table S4  *C. canimorsus* strain 5 surface-exposed lipoproteins. The footnotes in Table S4 follow. Footnote *a* indicates that using the annotated translational start site, Ccan_17430 is predicted to be a cytoplasmic protein, but if translation begins at an AUG 13 codons downstream, then it is predicted to be a lipoprotein. Footnote *b* indicates that using the annotated translational start site, Ccan_20120 is predicted to be a cytoplasmic protein, but if translation begins at an AUG 18 codons downstream, then it is predicted to be a lipoprotein. Footnote *c* indicates the SPII cleavage site predicted by the LipoP software; the numbers indicate the positions of the last amino acid of the signal peptide and the position of the +1 cysteine. Footnote *d* indicates the quantitative contribution to surfome composition, expressed as a percentage, as described in reference 17. Values that were not quantified are indicated (/). Table S4, DOCX file, 0.1 MB

Table S5  *Bacteroidetes* outer membrane lipoproteins. Footnote *a* indicates the SPII cleavage site predicted by the LipoP software. The numbers indicate the positions of the last amino acid of the signal peptide and the position of the +1 cysteine. Footnote *b* indicates that as described in reference 44, the translational start site of BF9343_1295 was moved 15 codons downstream, resulting in a predicted lipoprotein. Footnote *c* indicates that as described in reference 44, the translational start site of BF9343_p20 was moved 38 codons downstream, resulting in a predicted lipoprotein. Table S5, DOCX file, 0.1 MB
