# Erratum for Lauber et al., Identification of a New Lipoprotein Export Signal in Gram-Negative Bacteria

**DOI:** 10.1128/mBio.02007-16

**Published:** 2016-12-13

**Authors:** Frédéric Lauber, Guy Richard Cornelis, Francesco Renzi

**Affiliations:** Département de Biologie, Unité de Recherche en Biologie des Microorganismes (URBM), Université de Namur, Namur, Belgium

## ERRATUM

Volume 7, no. 5, doi:10.1128/mBio.01232-16, 2016. In the Results section (PDF page 5), in Fig. 3, panel D, the microscopy images of strain referenced as #5 were duplicates of the strain referenced as #4. The revised Fig. 3 (below) shows the correct microscopy images.

**FIG 3:**
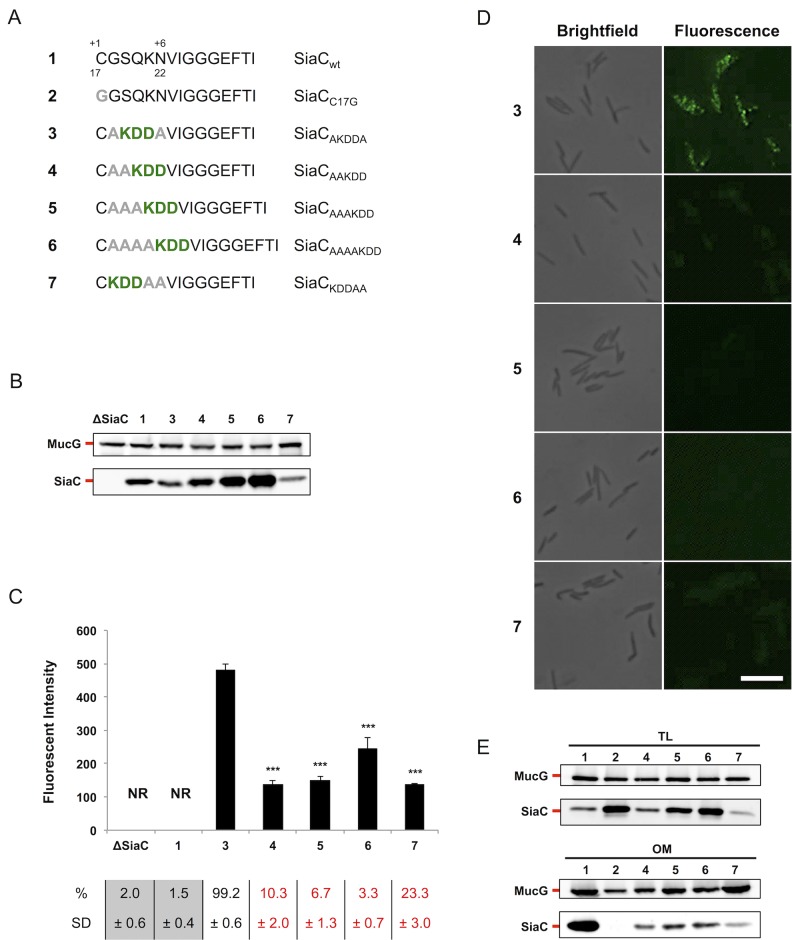
The position of the minimal LES is crucial for its function. (A) wt SiaC and consensus sequence mutant constructs. Amino acids derived from the consensus sequence (green boldface) and point mutations (grey boldface) are indicated. The SiaC constructs are referred to by the boldface numbers shown in panel A in panels B to E. (B) Detection of SiaC by Western blot analysis of total cell extracts of strains expressing the SiaC constructs shown in panel A. MucG expression was monitored as a loading control. (C) Quantification of SiaC surface exposure by flow cytometry of live cells labeled with anti-SiaC serum. The fluorescence intensity of stained cells only is shown (NR, not relevant). The averages from at least three independent experiments are shown. Error bars represent 1 standard deviation from the mean. Values that are significantly different (*P* ≤ 0.001) from the value for reference construct 3 are indicated (***). The percentage and standard deviation (SD) of stained cells are indicated below the bar graph. Values below the detection limit (≤2.5%) are shown on gray background. Values for strains with a statistically significant lower stained population are shown in red (*P* ≤ 0.001 compared to the value for the reference construct 3). (D) Immunofluorescence microscopy images of bacteria stained with anti-SiaC serum. Bar, 5 µm. (E) Western blot analysis of total lysates (TL) and outer membrane (OM) fractions of bacteria expressing different SiaC constructs. MucG expression was monitored as a loading control.

