# Supplementary figures and images for: Identification of a New Lipoprotein Export Signal in Gram-Negative Bacteria
Source: mBio. 2016 Oct 25;7(5):e01232-16. doi: 10.1128/mBio.01232-16 (PMC5080379; doi:10.1128/mBio.01232-16)

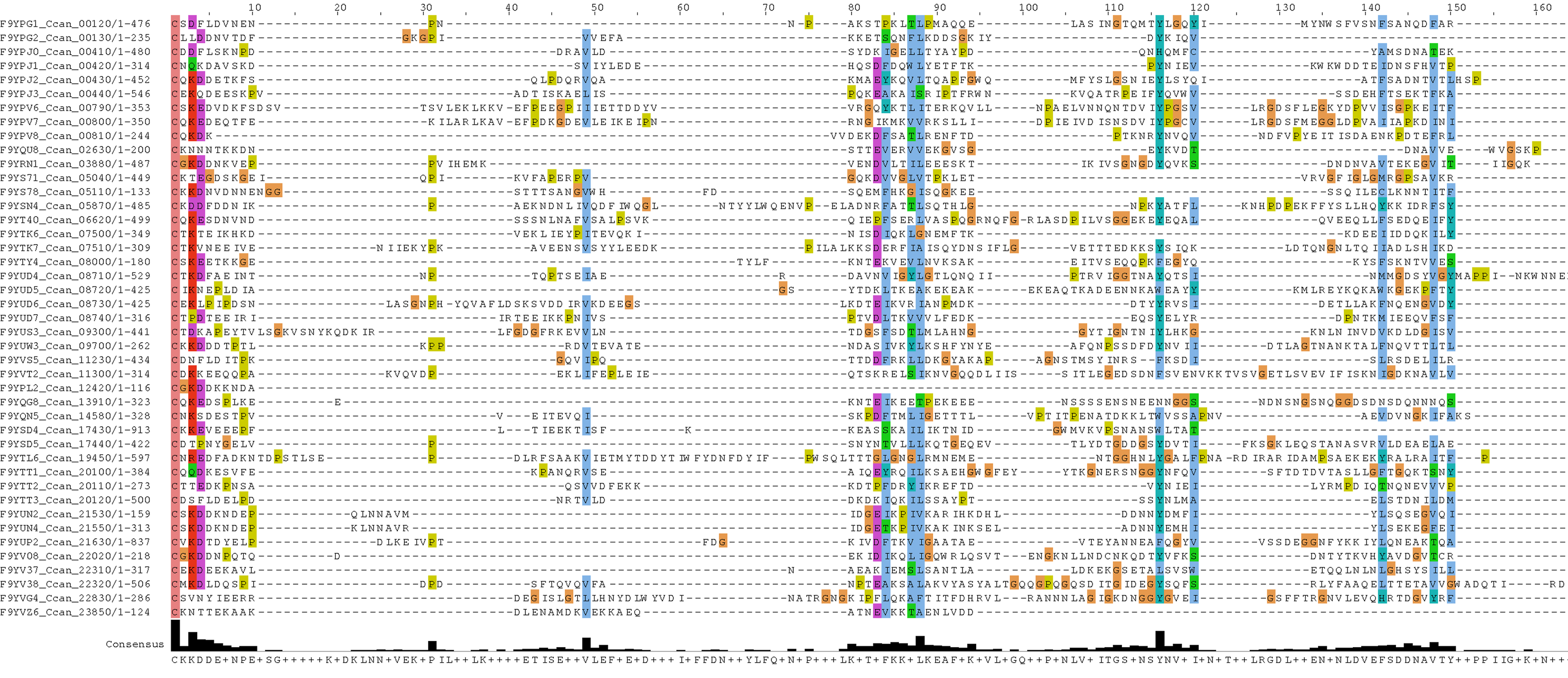

Supplement: Figure S1 — Multiple-sequence alignment of full-length C. canimorsus surface lipoproteins. MAFFT alignment was performed on mature surface-exposed lipoproteins. Only the N-terminal region showing the conserved K-(D/E) motif is displayed. Highly conserved residues are indicated according to the Clustal color code (R and K in red; D and E in magenta; P in yellow; G in orange; Q, N, S, and T in green; C in pink; A, I, L, M, F, W, and V in blue; H and Y in cyan). The derived consensus sequence is shown below the alignment. Download [file mbo005163032sf1.tif]

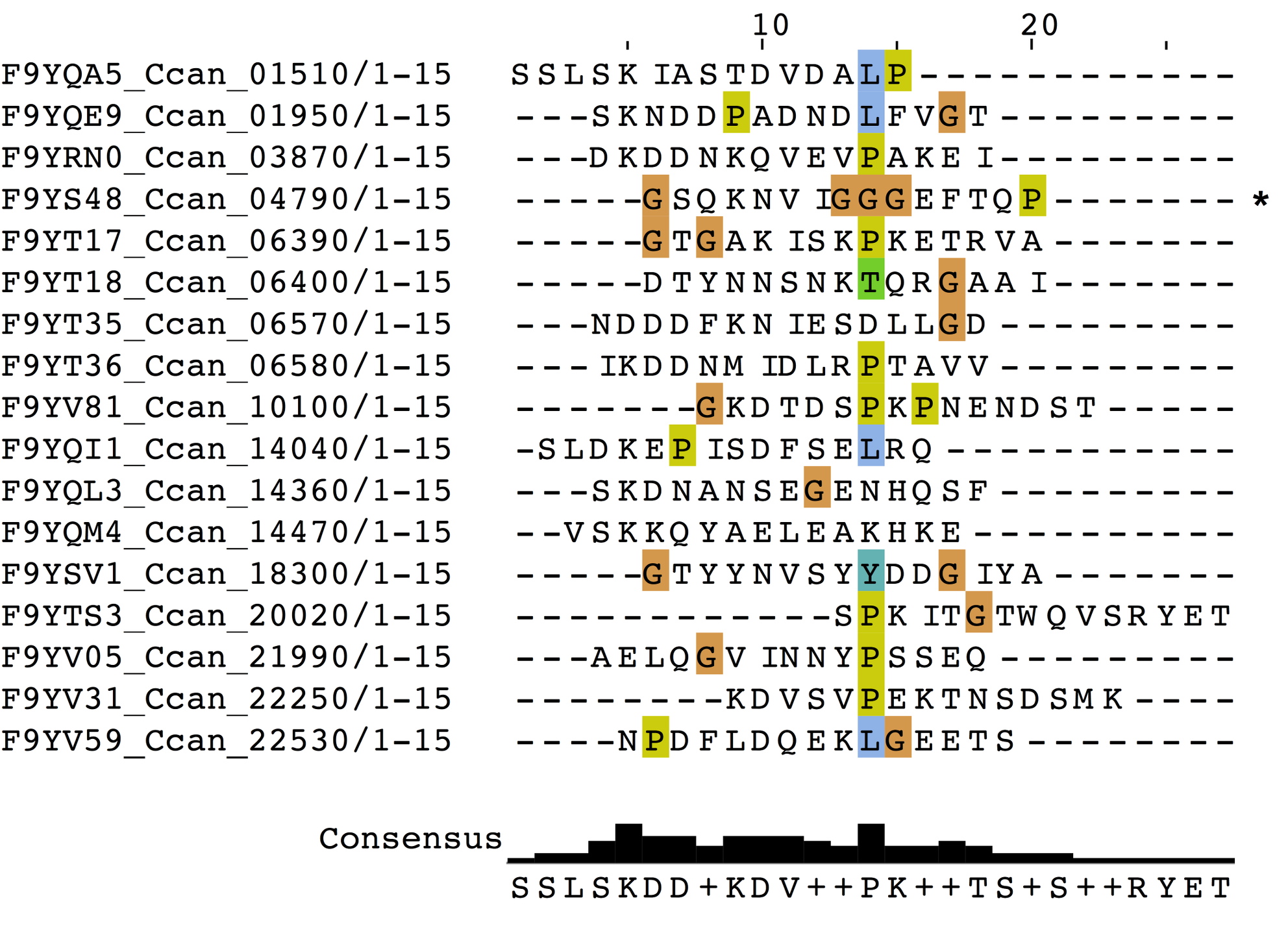

Supplement: Figure S2 — Multiple-sequence alignment of C. canimorsus periplasmic outer membrane lipoproteins. MAFFT alignment was performed on the first 15 N-terminal amino acids of intracellular OM lipoproteins. The first invariant cysteine residue of each sequence was removed before the alignment was performed. Highly conserved residues are indicated according to the Clustal color code (see the legend to Fig. S1 in the supplemental material). The derived consensus sequence is shown below. SiaC (Ccan_04790) is indicated by an asterisk. Download [file mbo005163032sf2.tif]

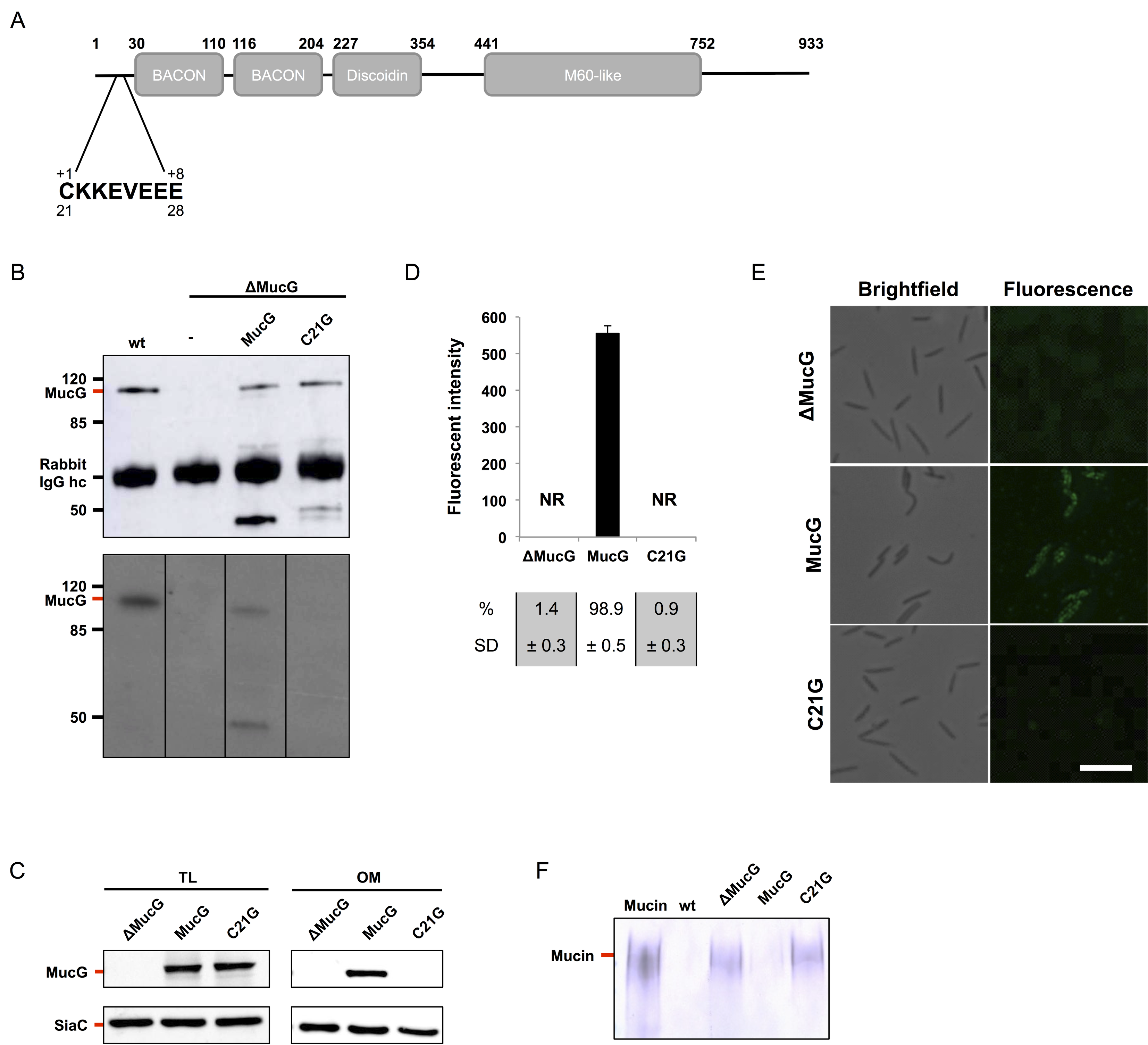

Supplement: Figure S3 — MucG is a surface-exposed lipoprotein. (A) MucG domain annotation. Predicted structural domains are indicated by gray boxes. Amino acid positions are indicated above the map. The predicted LES is shown below the map. (B) Western blot analysis (top) and fluorography (bottom) of the elution fraction of MucG immunoprecipitation of [3H]palmitate-labeled bacteria. MucG is lipidated in the wt strain and the ΔmucG strain expressing MucG, but not in the ΔmucG strain expressing MucGC21G in which the predicted site of lipidation is mutated, showing that MucG is a lipoprotein. Rabbit IgG hc is the heavy chain of the rabbit MucG antiserum present in the analyzed elution fraction. The low-molecular-weight band in the MucG strain likely represents a truncated MucG form due to overexpression. This band being radiolabeled indicates that the truncation takes place at the C terminus of MucG. The two low-molecular-weight bands in the MucGC21G mutant likely represent two different MucG truncated forms that are generated when the protein overexpressed is not lipidated and periplasmic. (C) MucG detection by Western blot analysis of total cell lysates (TL) and outer membrane (OM) fractions of bacteria expressing different MucG constructs. MucG, but not the soluble MucGC21G, is detected in the OM fraction, showing that MucG is a bona fide OM lipoprotein. SiaC expression was monitored as a loading control. (D) Quantification of MucG surface exposure by flow cytometry of live cells labeled with anti-MucG serum. The fluorescence intensity of stained cells only is shown (NR, not relevant). The averages from at least three independent experiments are shown. Error bars represent 1 standard deviation from the mean. The percentage and standard deviation (SD) of stained cells are indicated below the bar graph. Values below the detection limit (≤2.5%) are shown on a gray background. (E) Immunofluorescence microscopy images of bacteria labeled with anti-MucG serum. Bar, 5 µm. (F) Detection of mucin b [file mbo005163032sf3.tif]

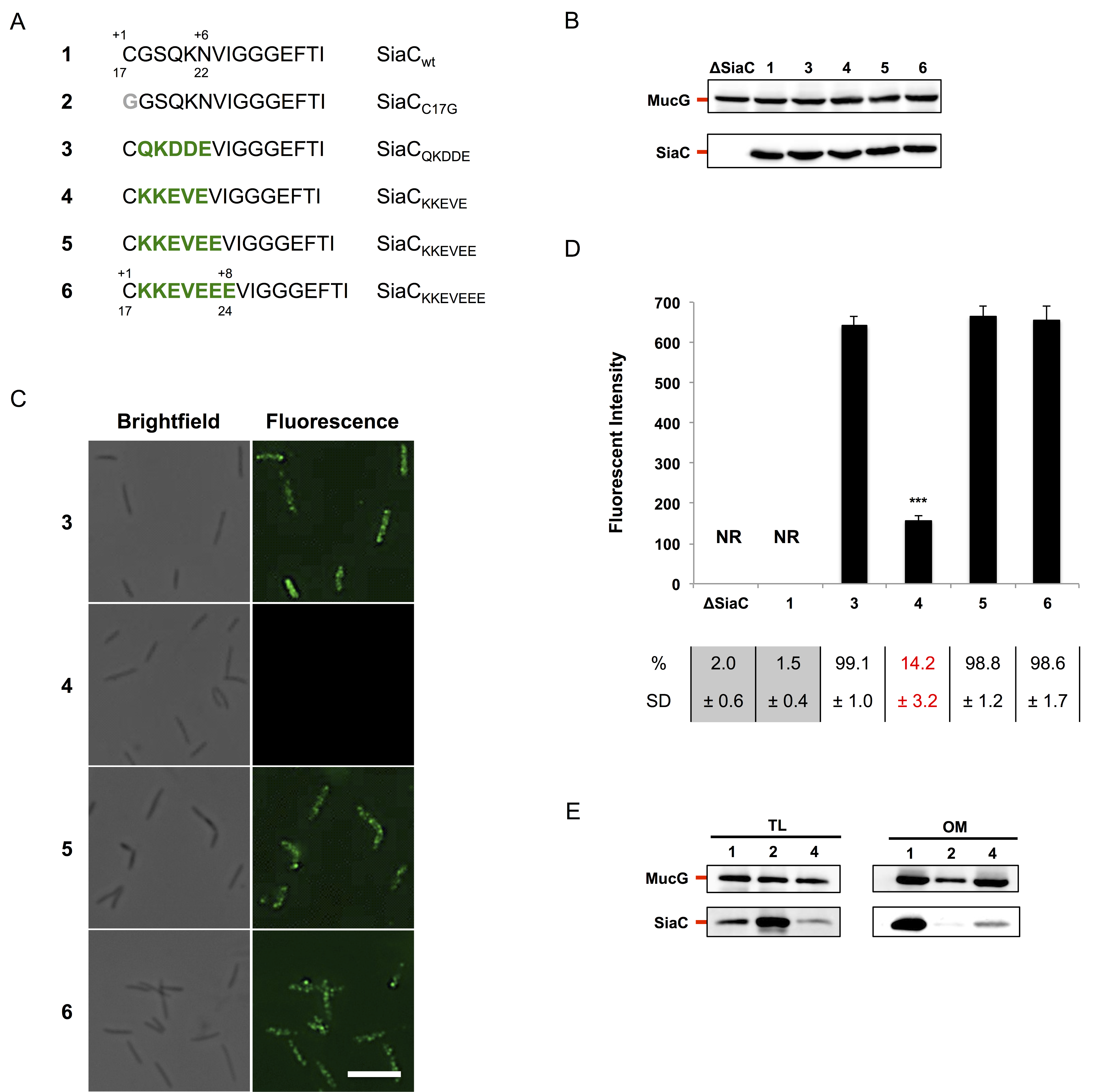

Supplement: Figure S4 — The MucG LES allows SiaC surface localization. (A) wt SiaC and MucG consensus sequence mutant constructs. Amino acids derived from the consensus or MucG LES (green boldface) and point mutations (gray boldface) are indicated. The SiaC constructs are referred to by the boldface numbers shown in panel A in panels B to E. (B) Detection of SiaC by Western blot analysis of total cell extracts of strains expressing the SiaC constructs shown in panel A. MucG expression was monitored as a loading control. (C) Immunofluorescence microscopy images of bacteria labeled with anti-SiaC serum. Bar, 5 µm. (D) Quantification of SiaC surface exposure by flow cytometry of live cells labeled with anti-SiaC serum. The fluorescence intensity of stained cells only is shown (NR, not relevant). The averages from at least three independent experiments are shown. Error bars represent 1 standard deviation from the mean. Values that are significantly different (P ≤ 0.001) from the value for reference construct 3 are indicated (***). The percentage and standard deviation (SD) of stained cells are indicated below the bar graph. Values below the detection limit (≤2.5%) are shown on a gray background, and values for strains with a statistically significant lower stained population are in red (P ≤ 0.001 compared to the value for reference construct 3). (E) Western blot analysis of total lysates (TL) and outer membrane (OM) fractions of bacteria expressing different SiaC constructs. MucG expression was monitored as a loading control. Download [file mbo005163032sf4.tif]

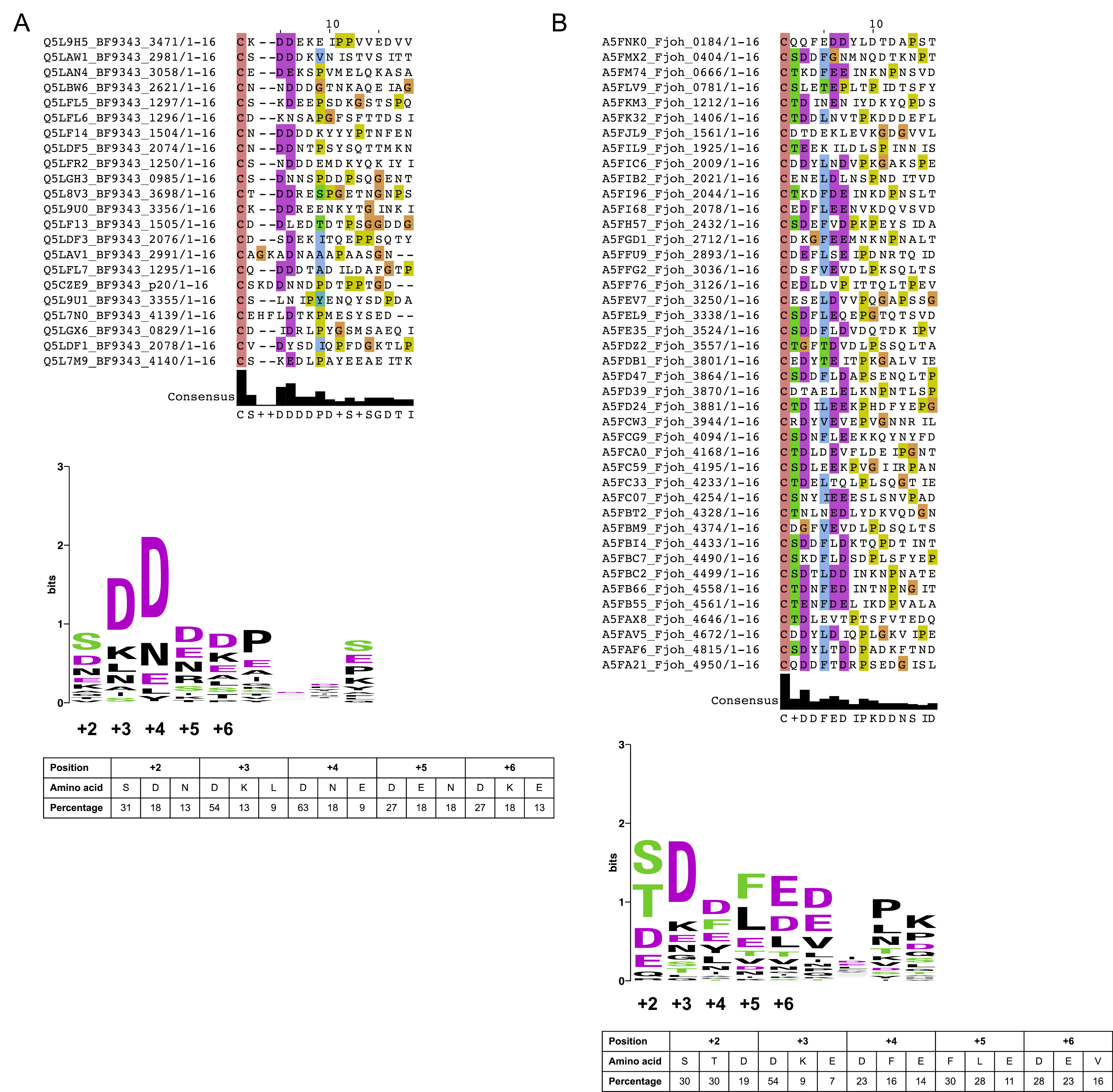

Supplement: Figure S5 — Multiple-sequence alignment of B. fragilis and F. johnsoniae surface lipoproteins. (A) MAFFT alignment of the first 16 N-terminal amino acids of proteinase K-sensitive B. fragilis lipoproteins. (B) MAFFT alignment of the first 16 N-terminal amino acids of SusD-like F. johnsoniae lipoproteins. Highly conserved residues are indicated according to the Clustal color code (see the legend to Fig. S1 in the supplemental material). Corresponding WebLogo and amino acid frequencies are indicated below. Download [file mbo005163032sf5.tif]
