# Supplementary material for: Identification of a New Lipoprotein Export Signal in Gram-Negative Bacteria
Source: mBio. 2016 Oct 25;7(5):e01232-16. doi: 10.1128/mBio.01232-16 (PMC5080379; doi:10.1128/mBio.01232-16)
Supplement: Table S1 — Bacterial strains used in this study. [file mbo005163032st1.docx]

**Table S1.** Bacterial strains used in this study

| **Strain** | **Genotype and/or description** | **Reference** |
| --- | --- | --- |
| *E. coli* | | |
| Top10 | F- *mcrA* Δ(*mrr-hsdRMS-mcrBC*) φ80*lac*ZΔM15 Δ*lacX*74 *recA1* *araD139* Δ(*araleu*)7697 *galU galK rpsL endA1 nupG*; Sm^r^ | Invitrogen |
| *C. canimorsus* | | |
| Cc5 | Wild type (BCCM-LMG 28512) | ([1](#_ENREF_1)) |
| Δ*siaC* | Replacement of *Ccan_04790* by *ermF*; Em^r^ | ([2](#_ENREF_2)) |
| Δ*mucG* | Replacement of *Ccan_17430* by *ermF*; Em^r^ | ([3](#_ENREF_3)) |

1. **Shin H, Mally M, Kuhn M, Paroz C, Cornelis GR.** 2007. Escape from immune surveillance by Capnocytophaga canimorsus. J Infect Dis **195:**375-386.

2. **Mally M, Cornelis GR.** 2008. Genetic tools for studying Capnocytophaga canimorsus. Appl Environ Microbiol **74:**6369-6377.

3. **Renzi F, Manfredi P, Dol M, Fu J, Vincent S, Cornelis GR.** 2015. Glycan-foraging systems reveal the adaptation of Capnocytophaga canimorsus to the dog mouth. MBio **6:**e02507.
