# Supplementary material for: Identification of a New Lipoprotein Export Signal in Gram-Negative Bacteria
Source: mBio. 2016 Oct 25;7(5):e01232-16. doi: 10.1128/mBio.01232-16 (PMC5080379; doi:10.1128/mBio.01232-16)
Supplement: Table S2 — Plasmids used in this study. Footnote a on Vectors indicates that selection markers for C. canimorsus are within parentheses. [file mbo005163032st2.docx]

**Table S2.** Plasmids used in this study

| **Plasmid** | **Description** | **Reference** |
| --- | --- | --- |
| **Vectors^a^** | | |
| pMM47.A | ColE1 *ori*; (pCC7 *ori*); Ap^r^; (Cfx^r^). *E. coli-C. canimorsus* expression shuttle plasmid with *ermF* promoter | ([1](#_ENREF_1)) |
| pPM5 | ColE1 *ori*; (pCC7 *ori*); Ap^r^; (Cfx^r^). *E. coli-C. canimorsus* expression shuttle plasmid with *ompA* promoter | ([2](#_ENREF_2)) |
| **Expression vectors** | | |
| pFL43 | Full lenght *mucG* with a C-terminal HA tag amplified with primers 7182/7625 and cloned into pPM5 using NcoI/XhoI restriction sites | This study |
| pFL44 | Full lenght *mucG* C21G with a C-terminal HA tag amplified with primers 7259/7625 and cloned into pPM5 using NcoI/XhoI restriction sites | This study |
| pFL71 | Full length *mucG* K22A with a C-terminal HA tag amplified with primers 7182/7487 and 7486/7625 and cloned into pPM5 using NcoI/XhoI restriction sites | This study |
| pFL72 | Full length *mucG* K23A with a C-terminal HA tag amplified with primers 7182/7489 and 7488/7625 and cloned into pPM5 using NcoI/XhoI restriction sites | This study |
| pFL73 | Full length *mucG* E24A with a C-terminal HA tag amplified with primers 7182/7491 and 7490/7625 and cloned into pPM5 using NcoI/XhoI restriction sites | This study |
| pFL74 | Full length *mucG* V25A with a C-terminal HA tag amplified with primers 7182/7493 and 7492/7625 and cloned into pPM5 using NcoI/XhoI restriction sites | This study |
| pFL75 | Full length *mucG* E26A with a C-terminal HA tag amplified with primers 7182/7495 and 7494/7625 and cloned into pPM5 using NcoI/XhoI restriction sites | This study |
| pFL76 | Full length *mucG* E27A with a C-terminal HA tag amplified with primers 7182/8048 and 8047/7625 and cloned into pPM5 using NcoI/XhoI restriction sites | This study |
| pFL77 | Full length *mucG* E28A with a C-terminal HA tag amplified with primers 7182/8050 and 8049/7625 and cloned into pPM5 using NcoI/XhoI restriction sites | This study |
| pFL79 | Full length *mucG* with a C-terminal HA tag amplified with primers 7182/7510 and 7509/7625 and cloned into pPM5 using NcoI/XhoI restriction sites. Replacement of aa 22-28 by AAEVEEE | This study |
| pFL84 | Full length *mucG* with a C-terminal HA tag amplified with primers 7182/7899 and 7898/7625 and cloned into pPM5 using NcoI/XhoI restriction sites. Replacement of aa 22-28 by KKAAAAA | This study |
| pFL117 | Full lenght *siaC* amplified with primers 4159 and 7696 and cloned into pMM47.A using NcoI/XhoI restriction sites | This study |
| pFL118 | Full lenght *siaC* C17G amplified with primers 5545 and 7696 and cloned into pMM47.A using NcoI/XhoI restriction sites | This study |
| pFL132 | Full lenght *siaC* amplified with primers 4159/8017 and 8016/7696 and cloned into pMM47.A using NcoI/XhoI restriction sites. Replacement of aa 18-22 by KKEVE | This study |
| pFL133 | Full lenght *siaC* amplified with primers 4159/8054 and 8052/7696 and cloned into pMM47.A using NcoI/XhoI restriction sites. Replacement of aa 18-22 by KKEVEE | This study |
| pFL134 | Full lenght *siaC* amplified with primers 4159/7972 and 7971/7696 and cloned into pMM47.A using NcoI/XhoI restriction sites. Replacement of aa 18-22 by KKEVEEE | This study |
| pFL143 | Full lenght *siaC* amplified with primers 4159/8058 and 8057/7696 and cloned into pMM47.A using NcoI/XhoI restriction sites. Replacement of aa 18-22 by QKDDE | This study |
| pFL144 | Full lenght *siaC* amplified with primers 4159/8086 and 8085/7696 and cloned into pMM47.A using NcoI/XhoI restriction sites. Replacement of aa 18-22 by AKDDE | This study |
| pFL145 | Full lenght *siaC* amplified with primers 4159/8084 and 8083/7696 and cloned into pMM47.A using NcoI/XhoI restriction sites. Replacement of aa 18-22 by AKDDA | This study |
| pFL146 | Full lenght *siaC* amplified with primers 4159/8153 and 8152/7696 and cloned into pMM47.A using NcoI/XhoI restriction sites. Replacement of aa 18-22 by AKEEA | This study |
| pFL147 | Full lenght *siaC* amplified with primers 4159/8149 and 8148/7696 and cloned into pMM47.A using NcoI/XhoI restriction sites. Replacement of aa 18-22 by AKDAA | This study |
| pFL148 | Full lenght *siaC* amplified with primers 4159/8151 and 8150/7696 and cloned into pMM47.A using NcoI/XhoI restriction sites. Replacement of aa 18-22 by AKEAA | This study |
| pFL149 | Full lenght *siaC* amplified with primers 4159/8157 and 8156/7696 and cloned into pMM47.A using NcoI/XhoI restriction sites. Replacement of aa 18-22 by AAKDD | This study |
| pFL150 | Full lenght *siaC* amplified with primers 4159/8159 and 8158/7696 and cloned into pMM47.A using NcoI/XhoI restriction sites. Replacement of aa 18-22 by AAAKDD | This study |
| pFL151 | Full lenght *siaC* amplified with primers 4159/8161 and 8160/7696 and cloned into pMM47.A using NcoI/XhoI restriction sites. Replacement of aa 18-22 by AAAAKDD | This study |
| pFL152 | Full lenght *siaC* amplified with primers 4159/8169 and 8168/7696 and cloned into pMM47.A using NcoI/XhoI restriction sites. Replacement of aa 18-22 by KDDAA | This study |
| pFL153 | Full lenght *siaC* amplified with primers 4159/8165 and 8164/7696 and cloned into pMM47.A using NcoI/XhoI restriction sites. Replacement of aa 18-22 by QADDE | This study |
| pFL154 | Full lenght *siaC* amplified with primers 4159/8167 and 8166/7696 and cloned into pMM47.A using NcoI/XhoI restriction sites. Replacement of aa 18-22 by AADDA | This study |
| pFL155 | Full lenght *siaC* amplified with primers 4159/8163 and 8162/7696 and cloned into pMM47.A using NcoI/XhoI restriction sites. Replacement of aa 18-22 by SDDFE | This study |
| pFL156 | Full lenght *siaC* amplified with primers 4159/8173 and 8172/7696 and cloned into pMM47.A using NcoI/XhoI restriction sites. Replacement of aa 18-22 by SDDDD | This study |

^a^: Selection markers for *C. canimorsus* are in between brackets

1. **Mally M, Cornelis GR.** 2008. Genetic tools for studying Capnocytophaga canimorsus. Appl Environ Microbiol **74:**6369-6377.

2. **Manfredi P, Lauber F, Renzi F, Hack K, Hess E, Cornelis GR.** 2015. New iron acquisition system in Bacteroidetes. Infect Immun **83:**300-310.
