# Supplementary material for: Identification of a New Lipoprotein Export Signal in Gram-Negative Bacteria
Source: mBio. 2016 Oct 25;7(5):e01232-16. doi: 10.1128/mBio.01232-16 (PMC5080379; doi:10.1128/mBio.01232-16)
Supplement: Table S3 — Oligonucleotides used in this study. Footnote a on Restriction indicates that the restriction sites are underlined in the sequence column. [file mbo005163032st3.docx]

**Table S3.** Oligonucleotides used in this study

| **Ref.** | **Sequence 5'-3'** | **Restriction^a^** |
| --- | --- | --- |
| 4159 | cataccatgggaaatcgaattttttatctt | NcoI |
| 5545 | catgccatgggaaatcgaattttttatcttttattcgcttttgttcttttgtcggctggtggaagccaaaaaaacg | NcoI |
| 7182 | ggccatggggaaaaaaatagtatccattagc | NcoI |
| 7259 | ggccatggggaaaaaaatagtatccattagcttatttttccttatctcagcaactatttggttagccggtaaaaaggaag | NcoI |
| 7625 | ggctcgagctaagcgtaatctggaacatcgtatgggtaaaacgtaacttgagttctc | XhoI |
| 7696 | ggctcgagttagttcttgataaattcctcaactgg | XhoI |
| 7486 | tggttagcctgtgcaaaggaagttgaagaagaacc |  |
| 7487 | ggttcttcttcaacttcctttgcacaggctaacca |  |
| 7488 | ttagcctgtaaagcggaagttgaagaagaaccttttc |  |
| 7489 | gaaaaggttcttcttcaacttccgctttacaggctaa |  |
| 7490 | gcctgtaaaaaggcagttgaagaagaaccttttctaac |  |
| 7491 | gttagaaaaggttcttcttcaactgcctttttacaggc |  |
| 7492 | tgtaaaaaggaagctgaagaagaaccttttctaac |  |
| 7493 | gttagaaaaggttcttcttcagcttcctttttaca |  |
| 7494 | aaaaaggaagttgcagaagaaccttttctaacaatag |  |
| 7495 | ctattgttagaaaaggttcttctgcaacttccttttt |  |
| 7509 | tggttagcctgtgcagcggaagttgaagaagaacc |  |
| 7510 | ggttcttcttcaacttccgctgcacaggctaacca |  |
| 7898 | gcagctgcagcggctccttttctaacaatagaagaaaaaacc |  |
| 7899 | agccgctgcagctgcctttttacaggctaaccaaatagttgc |  |
| 7971 | aaaaaggaagttgaagaagaagtaatcggcggaggcgaatttacacaacccg |  |
| 7972 | ttcttcttcaacttcctttttacaagccgacaaaagaacaaaagcg |  |
| 8016 | aaaaaggaagttgaagtaatcggcggaggcgaatttacacaacccg |  |
| 8017 | ttcaacttcctttttacaagccgacaaaagaacaaaagcg |  |
| 8047 | aggaagttgaagcagaaccttttctaacaatagaagaaaaaacc |  |
| 8048 | gaaaaggttctgcttcaacttcctttttacaggctaacc |  |
| 8049 | ggaagttgaagaagcaccttttctaacaatagaagaaaaaacc |  |
| 8050 | gaaaaggtgcttcttcaacttcctttttacaggctaaccaaatagttg |  |
| 8052 | aaaaaggaagttgaagaagtaatcggcggaggcgaatttacacaacccg |  |
| 8054 | ttcttcaacttcctttttacaagccgacaaaagaacaaaagcg |  |
| 8057 | caaaaggacgatgaagtaatcggcggaggcgaatttacacaacccg |  |
| 8058 | ttcatcgtccttttgacaagccgacaaaagaacaaaagcg |  |
| 8083 | gcaaaggacgatgcagtaatcggcggaggcgaatttacacaacccg |  |
| 8084 | tgcatcgtcctttgcacaagccgacaaaagaacaaaagcg |  |
| 8085 | gcaaaggacgatgaagtaatcggcggaggcgaatttacacaacccg |  |
| 8086 | ttcatcgtcctttgcacaagccgacaaaagaacaaaagcg |  |
| 8148 | gcaaaggacgctgcagtaatcggcggaggcgaatttacacaacccg |  |
| 8149 | tgcagcgtcctttgcacaagccgacaaaagaacaaaagcg |  |
| 8150 | gcaaaggaagctgcagtaatcggcggaggcgaatttacacaacccg |  |
| 8151 | tgcagcttcctttgcacaagccgacaaaagaacaaaagcg |  |
| 8152 | gcaaaggaagaggcagtaatcggcggaggcgaatttacacaacccg |  |
| 8153 | tgcctcttcctttgcacaagccgacaaaagaacaaaagcg |  |
| 8156 | gctgcaaaggacgatgtaatcggcggaggcgaatttacacaacccg |  |
| 8157 | atcgtcctttgcagcacaagccgacaaaagaacaaaagcg |  |
| 8158 | gcagctgcaaaggacgatgtaatcggcggaggcgaatttacacaacccg |  |
| 8159 | atcgtcctttgcagctgcacaagccgacaaaagaacaaaagcg |  |
| 8160 | gccgcagctgcaaaggacgatgtaatcggcggaggcgaatttacacaacccg |  |
| 8161 | atcgtcctttgcagctgcggcacaagccgacaaaagaacaaaagcg |  |
| 8162 | tctgatgacttcgaagtaatcggcggaggcgaatttacacaacccg |  |
| 8163 | ttcgaagtcatcagaacaagccgacaaaagaacaaaagcg |  |
| 8164 | caagcggacgatgaagtaatcggcggaggcgaatttacacaacccg |  |
| 8165 | ttcatcgtccgcttgacaagccgacaaaagaacaaaagcg |  |
| 8166 | gcagctgacgatgcagtaatcggcggaggcgaatttacacaacccg |  |
| 8167 | tgcatcgtcagctgcacaagccgacaaaagaacaaaagcg |  |
| 8168 | aaggacgatgcagctgtaatcggcggaggcgaatttacacaacccg |  |
| 8169 | agctgcatcgtccttacaagccgacaaaagaacaaaagcg |  |
| 8172 | agtgatgacgacgatgtaatcggcggaggcgaatttacacaacccg |  |
| 8173 | atcgtcgtcatcactacaagccgacaaaagaacaaaagcg |  |

^a^: Restriction sites are underlined
