# Supplementary material for: Identification of a New Lipoprotein Export Signal in Gram-Negative Bacteria
Source: mBio. 2016 Oct 25;7(5):e01232-16. doi: 10.1128/mBio.01232-16 (PMC5080379; doi:10.1128/mBio.01232-16)
Supplement: Table S4 — C. canimorsus strain 5 surface-exposed lipoproteins. The footnotes in Table S4 follow. Footnote a indicates that using the annotated translational start site, Ccan_17430 is predicted to be a cytoplasmic protein, but if translation begins at an AUG 13 codons downstream, then it is predicted to be a lipoprotein. Footnote b indicates that using the annotated translational start site, Ccan_20120 is predicted to be a cytoplasmic protein, but if translation begins at an AUG 18 codons downstream, then it is predicted to be a lipoprotein. Footnote c indicates the SPII cleavage site predicted by the LipoP software; the numbers indicate the positions of the last amino acid of the signal peptide and the position of the +1 cysteine. Footnote d indicates the quantitative contribution to surfome composition, expressed as a percentage, as described in reference 17. Values that were not quantified are indicated (/). [file mbo005163032st4.docx]

**Table S4.** *C. canimorsus* 5 surface exposed lipoproteins

| **Uniprot Accession** | **ORF name** | **Annotation** | **SPII cleavage site^c^** | **% of surfome^d^** |
| --- | --- | --- | --- | --- |
| F9YPG1 | Ccan_00120 | Uncharacterized protein | 22-23 | 8,35 |
| F9YPG2 | Ccan_00130 | Uncharacterized protein | 19-20 | 4,25 |
| F9YPJ0 | Ccan_00410 | Uncharacterized protein | 18-19 | 0,23 |
| F9YPJ1 | Ccan_00420 | Uncharacterized protein | 17-18 | 0,32 |
| F9YPJ2 | Ccan_00430 | Uncharacterized protein | 20-21 | 0,27 |
| F9YPJ3 | Ccan_00440 | Uncharacterized protein | 19-20 | 0,14 |
| F9YPV6 | Ccan_00790 | Uncharacterized protein | 19-20 | 12,8 |
| F9YPV7 | Ccan_00800 | Tetanolysin O | 19-20 | 0,58 |
| F9YPV8 | Ccan_00810 | Uncharacterized protein | 12-13 | 0,46 |
| F9YQU8 | Ccan_02630 | UPF0312 protein | 19-20 | 3,63 |
| F9YRN1 | Ccan_03880 | TvBspA-like-625 | 20-21 | 1,24 |
| F9YS71 | Ccan_05040 | Glycosyl hydrolase family 109 protein 5 (EC 3.2.1.49) | 26-27 | / |
| F9YS78 | Ccan_05110 | Uncharacterized protein | 18-19 | 0,69 |
| F9YSN4 | Ccan_05870 | Carboxyl-terminal-processing protease (EC 3.4.21.102) | 16-17 | 1,02 |
| F9YT40 | Ccan_06620 | Thiol-activated cytolysin | 21-22 | 1,37 |
| F9YTK6 | Ccan_07500 | Uncharacterized protein | 16-17 | 0,45 |
| F9YTK7 | Ccan_07510 | Uncharacterized protein | 15-16 | 0,2 |
| F9YTY4 | Ccan_08000 | Uncharacterized protein | 19-20 | / |
| F9YUD4 | Ccan_08710 | GpdD | 16-17 | 3,99 |
| F9YUD5 | Ccan_08720 | GpdG | 20-21 | 3,43 |
| F9YUD6 | Ccan_08730 | GpdE | 16-17 | 1,28 |
| F9YUD7 | Ccan_08740 | GpdF | 17-18 | 3,25 |
| F9YUS3 | Ccan_09300 | Thioredoxin family protein (EC 1.8.1.8) | 16-17 | / |
| F9YUW3 | Ccan_09700 | Peptidyl-prolyl cis-trans isomerase (EC 5.2.1.8) | 19-20 | 0,71 |
| F9YVS5 | Ccan_11230 | Uncharacterized protein | 17-18 | 0,17 |
| F9YVT2 | Ccan_11300 | Uncharacterized protein | 17-18 | 1,11 |
| F9YPL2 | Ccan_12420 | Uncharacterized protein | 18-19 | 2,57 |
| F9YQG8 | Ccan_13910 | Uncharacterized protein | 21-22 | 0,27 |
| F9YQN5 | Ccan_14580 | Internalin-J (EC 3.2.1.83) | 23-24 | 0,23 |
| F9YSD4 | Ccan_17430^a^ | MucG mucinase | 20-21 | 1,29 |
| F9YSD5 | Ccan_17440 | MucE | 18-19 | 8,99 |
| F9YTL6 | Ccan_19450 | Uncharacterized protein | 18-19 | 5,15 |
| F9YTT1 | Ccan_20100 | Uncharacterized protein | 19-20 | / |
| F9YTT2 | Ccan_20110 | Uncharacterized protein | 20-21 | 1,64 |
| F9YTT3 | Ccan_20120^b^ | Uncharacterized protein | 20-21 | 2,08 |
| F9YUN2 | Ccan_21530 | Uncharacterized protein | 23-24 | / |
| F9YUN4 | Ccan_21550 | Uncharacterized protein | 23-24 | 0,09 |
| F9YUP2 | Ccan_21630 | Uncharacterized protein | 24-25 | 11,3 |
| F9YV08 | Ccan_22020 | Uncharacterized protein | 17-18 | 0,03 |
| F9YV37 | Ccan_22310 | Uncharacterized protein | 21-22 | 0,17 |
| F9YV38 | Ccan_22320 | Uncharacterized protein | 20-21 | 0,19 |
| F9YVG4 | Ccan_22830 | Uncharacterized protein | 16-17 | 0,12 |
| F9YVZ6 | Ccan_23850 | Uncharacterized protein | 17-18 | / |
|  |  |  | **Total** | **84,06** |

^a^: Using the annotated translational start site Ccan_17430 is predicted to be a cytoplasmic protein, but if translation begins at an AUG 13 codons downstream then it is predicted to be a lipoprotein.

^b^: Using the annotated translational start site Ccan_20120 is predicted to be a cytoplasmic protein, but if translation begins at an AUG 18 codons downstream then it is predicted to be a lipoprotein.

^c^: SPII cleavage site predicted by the LipoP software; numbers indicate the position of the last amino acid of the signal peptide and the position of the +1 cysteine.

^d^: Quantitative contribution to surfome composition, expressed in percentage, as described in ([1](#_ENREF_1)).

‘/’ stands for not quantified.

1. **Manfredi P, Renzi F, Mally M, Sauteur L, Schmaler M, Moes S, Jeno P, Cornelis GR.** 2011. The genome and surface proteome of Capnocytophaga canimorsus reveal a key role of glycan foraging systems in host glycoproteins deglycosylation. Mol Microbiol **81:**1050-1060.
