# Supplementary material for: Identification of a New Lipoprotein Export Signal in Gram-Negative Bacteria
Source: mBio. 2016 Oct 25;7(5):e01232-16. doi: 10.1128/mBio.01232-16 (PMC5080379; doi:10.1128/mBio.01232-16)
Supplement: Table S5 — Bacteroidetes outer membrane lipoproteins. Footnote a indicates the SPII cleavage site predicted by the LipoP software. The numbers indicate the positions of the last amino acid of the signal peptide and the position of the +1 cysteine. Footnote b indicates that as described in reference 44, the translational start site of BF9343_1295 was moved 15 codons downstream, resulting in a predicted lipoprotein. Footnote c indicates that as described in reference 44, the translational start site of BF9343_p20 was moved 38 codons downstream, resulting in a predicted lipoprotein. [file mbo005163032st5.docx]

**Table S5.** Bacteroidetes outer membrane lipoproteins

| **Uniprot Accession** | **ORF name** | **Annotation** | **SPII cleavage site^a^** |
| --- | --- | --- | --- |
| ***C. canimorsus* 5 periplasmic outer membrane lipoproteins** | | | |
| F9YQA5 | Ccan_01510 | Putative Subtilisin (EC 3.4.21.62) | 18-19 |
| F9YQE9 | Ccan_01950 | Uncharacterized protein | 19-20 |
| F9YRN0 | Ccan_03870 | Surface antigen BspA | 20-21 |
| F9YS48 | Ccan_04790 | Neuraminidase | 16-17 |
| F9YT17 | Ccan_06390 | Membrane or secreted protein | 15-16 |
| F9YT18 | Ccan_06400 | Inner membrane lipoprotein yiaD | 16-17 |
| F9YT35 | Ccan_06570 | Uncharacterized protein | 19-20 |
| F9YT36 | Ccan_06580 | Uncharacterized protein | 22-23 |
| F9YV81 | Ccan_10100 | Uncharacterized protein | 19-20 |
| F9YQI1 | Ccan_14040 | Uncharacterized protein | 16-17 |
| F9YQL3 | Ccan_14360 | Uncharacterized protein | 32-33 |
| F9YQM4 | Ccan_14470 | OmpA/MotB C-terminal like outer membrane protein | 17-18 |
| F9YSV1 | Ccan_18300 | Uncharacterized protein | 25-26 |
| F9YTS3 | Ccan_20020 | Uncharacterized protein | 20-21 |
| F9YV05 | Ccan_21990 | Uncharacterized protein | 16-17 |
| F9YV31 | Ccan_22250 | TvaII (EC 3.2.1.1) | 36-37 |
| F9YV59 | Ccan_22530 | Uncharacterized protein | 20-21 |
| ***B. fragilis* NCTC 9343 proteinase K sensitive surface exposed lipoproteins** | | | |
| Q5L9H5 | BF9343_3471 | Uncharacterized protein | 21-22 |
| Q5LAW1 | BF9343_2981 | Putative lipoprotein | 22-23 |
| Q5LAN4 | BF9343_3058 | Putative lipoprotein | 18-19 |
| Q5LBW6 | BF9343_2621 | Putative lipoprotein | 22-23 |
| Q5LFL5 | BF9343_1297 | Uncharacterized protein | 18-19 |
| Q5LFL6 | BF9343_1296 | Uncharacterized protein | 20-21 |
| Q5LF14 | BF9343_1504 | Uncharacterized protein | 25-26 |
| Q5LDF5 | BF9343_2074 | Putative exported protein | 21-22 |
| Q5LFR2 | BF9343_1250 | Uncharacterized protein | 22-23 |
| Q5LGH3 | BF9343_0985 | Conserved hypothetical lipoprotein | 24-25 |
| Q5L8V3 | BF9343_3698 | Putative exported protein | 20-21 |
| Q5L9U0 | BF9343_3356 | Putative lipoprotein | 23-24 |
| Q5LF13 | BF9343_1505 | Uncharacterized protein | 37-38 |
| Q5LDF3 | BF9343_2076 | Putative lipoprotein | 25-26 |
| Q5LAV1 | BF9343_2991 | Putative exported protein | 19-20 |
| Q5LFL7 | BF9343_1295^b^ | Uncharacterized protein | 24-25 |
| Q5CZE9 | BF9343_p20^c^ | Uncharacterized protein | 18-19 |
| Q5L9U1 | BF9343_3355 | Uncharacterized protein | 29-30 |
| Q5L7N0 | BF9343_4139 | Putative outer membrane protein | 28-29 |
| Q5LGX6 | BF9343_0829 | Possible outer membrane protein | 16-17 |
| Q5LDF1 | BF9343_2078 | Conserved hypothetical lipoprotein | 21-22 |
| Q5L7M9 | BF9343_4140 | Uncharacterized protein | 25-26 |
| ***F. johnsoniae* UW101 SusD-like lipoproteins** | | | |
| A5FNK0 | Fjoh_0184 | RagB/SusD domain protein | 22-23 |
| A5FMX2 | Fjoh_0404 | RagB/SusD domain protein | 17-18 |
| A5FM74 | Fjoh_0666 | RagB/SusD domain protein | 19-20 |
| A5FLV9 | Fjoh_0781 | RagB/SusD domain protein | 26-27 |
| A5FKM3 | Fjoh_1212 | RagB/SusD domain protein | 19-20 |
| A5FK32 | Fjoh_1406 | RagB/SusD domain protein | 24-25 |
| A5FJL9 | Fjoh_1561 | RagB/SusD domain protein | 21-22 |
| A5FIL9 | Fjoh_1925 | RagB/SusD domain protein | 19-20 |
| A5FIC6 | Fjoh_2009 | RagB/SusD domain protein | 20-21 |
| A5FIB2 | Fjoh_2021 | RagB/SusD domain protein | 18-19 |
| A5FI96 | Fjoh_2044 | RagB/SusD domain protein | 22-23 |
| A5FI68 | Fjoh_2078 | RagB/SusD domain protein | 20-21 |
| A5FH57 | Fjoh_2432 | RagB/SusD domain protein | 21-22 |
| A5FGD1 | Fjoh_2712 | RagB/SusD domain protein | 21-22 |
| A5FFU9 | Fjoh_2893 | RagB/SusD domain protein | 18-19 |
| A5FFG2 | Fjoh_3036 | RagB/SusD domain protein | 34-35 |
| A5FF76 | Fjoh_3126 | RagB/SusD domain protein | 20-21 |
| A5FEV7 | Fjoh_3250 | RagB/SusD domain protein | 19-20 |
| A5FEL9 | Fjoh_3338 | RagB/SusD domain protein | 24-25 |
| A5FE35 | Fjoh_3524 | RagB/SusD domain protein | 17-18 |
| A5FDZ2 | Fjoh_3557 | RagB/SusD domain protein | 27-28 |
| A5FDB1 | Fjoh_3801 | RagB/SusD domain protein | 18-19 |
| A5FD47 | Fjoh_3864 | RagB/SusD domain protein | 21-22 |
| A5FD39 | Fjoh_3870 | RagB/SusD domain protein | 20-21 |
| A5FD24 | Fjoh_3881 | RagB/SusD domain protein | 21-22 |
| A5FCW3 | Fjoh_3944 | RagB/SusD domain protein | 19-20 |
| A5FCG9 | Fjoh_4094 | RagB/SusD domain protein | 20-21 |
| A5FCA0 | Fjoh_4168 | RagB/SusD domain protein | 23-24 |
| A5FC59 | Fjoh_4195 | RagB/SusD domain protein | 17-18 |
| A5FC33 | Fjoh_4233 | RagB/SusD domain protein | 21-22 |
| A5FC07 | Fjoh_4254 | RagB/SusD domain protein | 17-18 |
| A5FBT2 | Fjoh_4328 | RagB/SusD domain protein | 18-19 |
| A5FBM9 | Fjoh_4374 | RagB/SusD domain protein | 34-35 |
| A5FBI4 | Fjoh_4433 | RagB/SusD domain protein | 20-21 |
| A5FBC7 | Fjoh_4490 | RagB/SusD domain protein | 24-25 |
| A5FBC2 | Fjoh_4499 | RagB/SusD domain protein | 17-18 |
| A5FB66 | Fjoh_4558 | RagB/SusD domain protein | 20-21 |
| A5FB55 | Fjoh_4561 | RagB/SusD domain protein | 18-19 |
| A5FAX8 | Fjoh_4646 | RagB/SusD domain protein | 17-19 |
| A5FAV5 | Fjoh_4672 | RagB/SusD domain protein | 19-20 |
| A5FAF6 | Fjoh_4815 | RagB/SusD domain protein | 25-26 |
| A5FA21 | Fjoh_4950 | RagB/SusD domain protein | 24-25 |

^a^: SPII cleavage site predicted by the LipoP software; numbers indicate the position of the last amino acid of the signal peptide and the position of the +1 cysteine.

^b^: As described in reference ([1](#_ENREF_1)), the translational start site of BF9343_1295 was moved 15 codons downstream, resulting in a predicted lipoprotein.

^c^: As described in reference ([1](#_ENREF_1)), the translational start site of BF9343_p20 was moved 38 codons downstream, resulting in a predicted lipoprotein.

1. **Wilson MM, Anderson DE, Bernstein HD.** 2015. Analysis of the outer membrane proteome and secretome of Bacteroides fragilis reveals a multiplicity of secretion mechanisms. PLoS One **10:**e0117732.
